# Noninvasive high-frequency oscillation ventilation as post- extubation respiratory support in neonates: Systematic review and meta-analysis

**DOI:** 10.1371/journal.pone.0307903

**Published:** 2024-07-30

**Authors:** Rameshwar Prasad, Bijan Saha, Md Habibullah Sk, Jagdish Prasad Sahoo, Bhupendra Kumar Gupta, Subhash Chandra Shaw

**Affiliations:** 1 Department of Neonatology, All India Institute of Medical Sciences, Patna, Bihar, India; 2 Department of Neonatology, Institute of Postgraduate Medical Education and Research, Kolkata, West Bengal, India; 3 Department of Neonatology, All India Institute of Medical Sciences, Bhubaneshwar, Odisha, India; 4 Apollo Sage Hospital, Bhopal, Madhya Pradesh, India; 5 Department of Pediatrics, Army Hospital Research and Referral, Delhi, New Delhi, India; Kobe University Graduate School of Medicine School of Medicine, JAPAN

## Abstract

**Introduction:**

Noninvasive High-Frequency Oscillatory Ventilation (NHFOV) is increasingly being adopted to reduce the need for invasive ventilation after extubation.

**Objectives:**

To evaluate the benefits and harms of NHFOV as post-extubation respiratory support in newborns compared to other non‐invasive respiratory support modes.

**Material & methods:**

We included randomized controlled trials comparing NHFOV with other non-invasive modes post-extubation in newborns. Data sources were MEDLINE (via Pubmed), Cochrane Central Register of Controlled Trials, China National Knowledge Infrastructure, WHO international clinical trials registry platform and Clinical Trial Registry, forward and backward citation search. Methodological quality of studies was assessed by Cochrane’s Risk of Bias tool 1.0.

**Results:**

This systematic review included 21 studies and 3294 participants, the majority of whom were preterm. NHFOV compared to nasal continuous positive airway pressure (NCPAP) reduced reintubation within seven days (RR 0.34, 95% CI 0.22 to 0.53) after extubation. It also reduced extubation failure (RR 0.39, 95% CI 0.30 to 0.51) and reintubation within 72 hrs (RR 0.40, 95% CI 0.31 to 0.53), bronchopulmonary dysplasia (RR 0.59, 95% CI 0.37 to 0.94) and pulmonary air leak (RR 0.46, 95% CI 0.27 to 0.79) compared to NCPAP. The rate of reintubation within seven days (RR 0.62, 95% CI 0.18 to 2.14) was similar whereas extubation failure (RR 0.65, 95% CI 0.50 to 0.83) and reintubation (RR 0.68, 95% CI 0.52 to 0.89) within 72 hrs were lower in NHFOV group compared to nasal intermittent positive pressure ventilation. There was no effect on other outcomes. Overall quality of the evidence was low to very low in both comparisons.

**Conclusions:**

NHFOV may reduce the rate of reintubation and extubation failure post-extubation without increasing complications. Majority of the trials were exclusively done in preterm neonates. Further research with high methodological quality is warranted.

## Background

The use of invasive mechanical ventilation (IMV) in neonates with severe respiratory disease may lead to complications such as air leaks, bronchopulmonary dysplasia (BPD), and impaired neurodevelopmental outcomes. This has prompted a strategy of early weaning and extubation.

Post-extubation strategies like non-invasive respiratory support (NRS) are employed to minimize reintubation and reduce extubation failure rates. Nevertheless, neonates may still require reintubation, which is an independent risk factor for increased mortality and morbidity [1].

High failure rates of nasal continuous positive airway pressure (NCPAP) as post-extubation NRS have led to the search for more effective non-invasive options. Nasal intermittent positive pressure ventilation (NIPPV) has shown promise in reducing extubation failure rates compared to NCPAP. NIPPV reduces chronic lung disease slightly although it has little impact on mortality compared to NCPAP [2].

Non-invasive high-frequency oscillatory ventilation (NHFOV) is a newer approach that combines the advantages of high-frequency oscillatory ventilation (HFOV) and nasal CPAP. NHFOV offers physiological advantages, including an increase in functional residual capacity leading to improved oxygenation, elimination of the need for synchronization, and efficient CO2 removal [3, 4].

The adoption of NHFOV is on the rise and is becoming more prevalent in neonatal intensive care units (NICUs) [5]. In a meta-analysis, neonates with respiratory distress syndrome had fewer intubations when using NHFOV as the primary respiratory support [6]. Systematic reviews suggest that NHFOV and NIPPV are more effective than CPAP in reducing neonatal morbidities [7, 8].

Previous systematic reviews that examined the efficacy of NHFOV as post-extubation respiratory support were focused on preterm neonates and suggested that NHFOV may lower the need for reintubation after extubation in mechanically ventilated newborns without notable adverse effects [9, 10]. However, the quality of evidence was found to be low to very low. Meta-analyses compared NHFOV only against NIPPV and CPAP for post-extubation respiratory support and only one among them compared NHFOV and CPAP. Furthermore, none of the previous meta-analyses examined the efficacy of NHFOV on the need for escalation to other NRS methods post-extubation. The effectiveness of NHFOV in term neonates for post-extubation respiratory support still remains uncertain. In light of the current gaps in knowledge, the objective of the present study was to conduct an exploratory review to synthesize all available evidence on the use of NHFOV in neonates.

### Objective

To evaluate the benefits and harms of NHFOV as post-extubation respiratory support in newborns compared to other forms of non‐invasive respiratory support.

## Methods

This systematic review was performed according to the Preferred Reporting Items for Systematic Reviews and Meta-Analyses guideline [11] and was registered in PROSPERO (CRD42022374609).

### Study selection

#### Inclusion criteria

All included studies fulfilled the following criteria:

#### Type of studies

Randomised, quasi-randomised and cluster randomised trials that evaluated at least one of the prespecified outcomes.

#### Type of population

Neonates of all gestation extubated to an NRS from invasive mechanical ventilation.

#### Type of intervention

The trials that compared NHFOV with any other form of NRS post-extubation. Eligible control interventions: NCPAP, NIPPV, synchronized NIPPV (SNIPPV), non-Invasive Ventilation-Neurally Adjusted Ventilatory Assist (NIV-NAVA), heated humidified high flow nasal cannula (HHHFNC), oxygen/or no treatment. Any interface or device used to deliver NRS was included.

### Outcomes

1. Primary outcome
  - Need for endotracheal re-intubation within seven days of extubation.
2. The secondary outcomes
  - All-cause mortality before hospital discharge.
  - All-cause mortality at 28 days.
  - Failure of extubation (reintubation plus escalation or crossover to another NRS mode as rescue treatment).
  - BPD (defined as need for oxygen or respiratory support at 36 weeks postmenstrual age).
  - Composite outcome of death/BPD.
  - Nasal injury.
  - Pulmonary air leak (pulmonary interstitial emphysema, pneumothorax. pneumomediastinum, pneumopericardium).
  - Intraventricular haemorrhage (IVH) (any and severe–grade ≥3) [12].
  - Length of hospital stay (LOS).
  - Retinopathy of prematurity (ROP) (any and severe—stage ≥3) as per the International Committee for the Classification of Retinopathy of Prematurity classification [13].

### Exclusion criteria

The exclusion criteria were as follows:

1. Observational studies, reviews, cross-over trial
2. Overlapping study
3. Non-clinical studies
4. Studies that enrolled neonates who were extubated immediately after surfactant administration were excluded
5. NHFOV post-surgery

### Search strategy

Electronic databases—MEDLINE (via Pubmed), Cochrane Central Register of Controlled Trials (CENTRAL), China National Knowledge Infrastructure (CNKI) and clinical trial registries (WHO international clinical trials registry platform and ClinicalTrials.gov) were searched. without any restriction on language, publication date, or publication status. Final search was done on 30th June 2023. The search strategy is given in **S1 Table.** The reference lists of publications eligible for full-text review and topic-related review articles were searched to identify additional studies. Further, Google Scholar was used to identify and screen reports citing eligible studies.

### Study selection

After deduplication, two independent reviewers screened the titles and abstracts of all records retrieved and excluded studies unrelated to our topic. Next, two researchers independently assessed the full-text articles of the selected records for their eligibility according to inclusion and exclusion criteria described above. Disagreements were resolved by discussion or by a third reviewer. Author names, trial registration numbers, study locations, dates and sample characteristics were used to recognize multiple reports originating from the same study **S1 Appendix.**

### Assessment of methodological quality

We assessed risk of bias in the included studies using the Cochrane Collaboration ‘Risk of bias’ tool for randomised trials (RoB 1.0) [14]. The following domains were assessed—selection bias, performance bias, detection bias, attrition bias, reporting bias, and other bias. Two review authors independently assessed the RoB. Disagreements were sorted out by discussion or by consulting the third reviewer **S1 Appendix.**

### Quality of evidence

We used Grading of Recommendations Assessment, Development, and Evaluation (GRADE) approach [15] to assess the quality of evidence for the following seven clinically relevant outcomes: 1. Reintubation within 7 days, 2. Extubation failure within 72 hrs 3. All-cause mortality at hospital discharge, 4. BPD, 5. Pulmonary air leak, 6. IVH severe–grade 3/4, 7. ROP -stage ≥3.

### Statistical analysis and data synthesis

The data analysis was conducted using Review Manager version 5.4.1 with a fixed-effect model. A random-effects model was employed when heterogeneity (I^2^) exceeded 50%. Risk Ratio (RR) was used for dichotomous outcomes, the standardized mean difference (SMD) for continuous data, and 95% confidence intervals (CI) were reported. For hospital stay duration reported as median and interquartile ranges, the mean was estimated using methods proposed by Wang et al. [16]. We analysed data on an intention-to-treat basis. We analysed data on all-trial level and separately for preterm subgroup. However, for BPD, ROP and IVH, data for only preterm neonates were pooled for primary analysis. The Number Needed to Treat for an additional beneficial outcome (NNTB) or harmful outcome (NNTH) was calculated for statistically significant results. Leave-one-out meta-analysis was done in RStudio version 4.2.1.

## Results

### Search results

**Fig 1:**
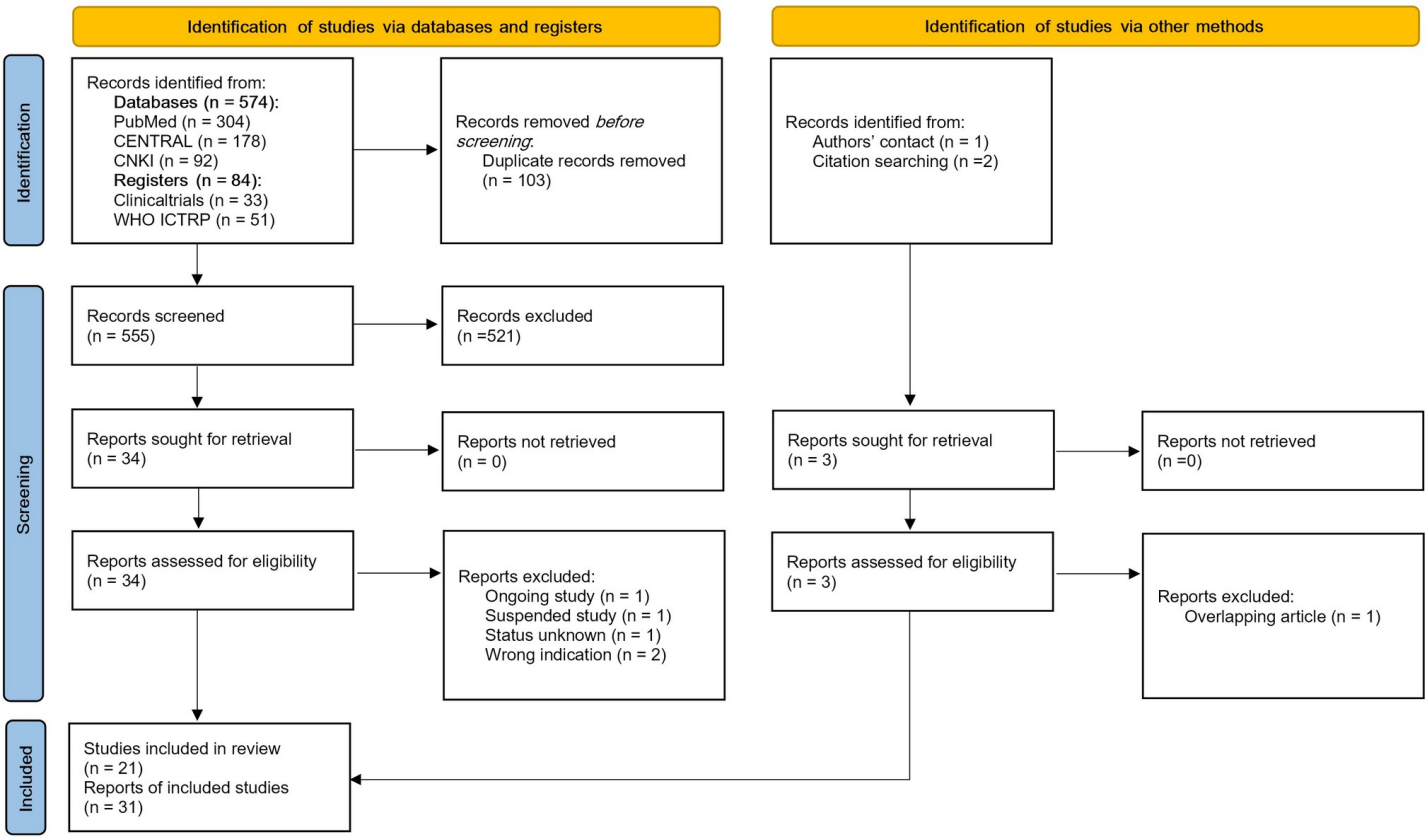
Flow diagram of the study selection process.

Electronic database search yielded 658 records. After removing 103 duplicate records, 521 records were excluded based on title and abstract screening. We assessed 34 reports which included 10 trial register reports (TRR)(CTRI//07/020055, NCT02340299, NCT03140891, NCT02543125, NCT04905732, NCT01852916, ChiCTR1900024289, NCT05493527, NCT03181958, NCT0432339), one published protocol [17] and 23 full text articles [18–40] for full-text retrieval and screening. Authors of two unpublished trials (NCT02543125, NCT05493527) were contacted. One of them (NCT05493527) responded and shared their full text article [41] which was included. Additionally, two more records were identified through citation searching and were included [42, 43]. Furthermore, additional relevant data were provided by Seth et al. [23] Finally, 31 reports (7 TRRs, 1 published protocol [17], 23 full-text articles [18–37, 41–43]) that represented 21 RCTs were included. The study selection process is shown in **Fig 1.** We listed excluded trials and reasons for their exclusion in **S2 Table**.

### Characteristics of included studies

Seventeen studies were from China [18, 21, 24, 26–37, 42, 43] and there was one each from Thailand [20], India [23], Germany [25] and Egypt [41]. Only one study was multicentric [18]. Additionally, twelve papers were exclusively available in the Chinese language and were translated into English using Google Translate [28–37, 42, 43]. There was one more article in Chinese with an available English translation [27]. We provided details in the Characteristics of included studies **Tables 1–3.**

**Table 1:** Characteristics of included studies (Studies comparing NHFOV vs NCPAP).

	Study ID	Country & center	Sample size Total(N)	Male, n(%)	Antenatal steroids, n(%)	Gestational age(weeks), mean±SD		Birth weight (g), mean±SD	
						NHFOV	NCPAP	NHFOV	NCPAP
1.	Chen 2019 [24]	China, single	N = 206 NHFOV = 103 NCPAP = 103	123(59.7)	185(89.8)	32.4 ±2.4	32.7±2.4	1859.1±569.1	1917.1±477.9
2.	Fischer 2019 [25]	Germany, single	N = 6 NHFOV = 4 NCPAP = 2	3(50)	NR	25 0/7 (23 4/7-6 3/7)^a^	24 0/3 (23 6/7-24 6/7)^a^	500(420–568)^a^	668(550 to 786)^a^
3.	Lou 2017a [27]	China, single	N = 69 NHFOV = 35 NCPAP = 34	48(69.6)	NR	39.32 ± 1.41	39.84 ± 1.22	3372.37 ± 431.20	3 396.25 ± 495.47
4.	Wang 2022 [35]	China, single	N = 74 NHFOV = 37 NCPAP = 37	39(52.7)	NR	32.61±1.52	32.78±1.87	2310±580	2360±560
5.	Wang 2020 [34]	China, single	N = 80 NHFOV = 40 NCPAP = 40	56(70)	NR	NR	NR	3330±240	3410±180
6.	Guo 2021 [36]	China, single	N = 60 NHFOV = 30 NCPAP = 30	29(48.3)	NR	NR	NR	2230 ± 630	2260 ± 710 kg
7.	Cao 2019 [33]	China, single	N = 60 NHFOV = 30 NCPAP = 30	33(55)	NR	36.1±0.2	35.9±0.1	3801.2±260.5	3825.2±274.5
8.	Lou 2017b [31]	China, single	N = 65 NHFOV = 34 NCPAP = 31	46(70.8)	25(38.5)	32.5 ± 1.3	32.4 ± 1.4	1790 ± 410	1850 ± 410
9.	Zhang 2021 [32]	China, single	N = 70 NHFOV = 35 NCPAP = 35	48(68.6)	64(91.4)	33.8±5.0.8	33.64±1.06	1984.57±245.75	1878.43±325.20

SD, standard deviation; NHFOV, non-invasive high frequency ventilation; NCPAP, nasal positive airway pressure; NIPPV, non-invasive positive pressure ventilation; NR, not reported.

^a^median (IQR)

**Table 2:** Characteristics of included studies (Studies comparing NHFOV vs NIPPV).

	Study ID	Country & center	Sample size Total(N)	Male,n(%)	Antenatal steroids, n(%)	Gestational age(weeks), mean±SD		Birth weight (g), mean±SD	
						NHFOV	NIPPV	NHFOV	NIPPV
1.	Seth 2021 [23]	India, single	N = 86 NHFOV = 43 NIPPV = 43	48 (55.8)	52(60.5)	32(28–35)^a^	31(29–35)^a^	1500 (1120–2140)^a^	1495 (980–2214)^a^
2.	Phatigomet 2023 [20]	Thailand, single	N = 133 NHFOV = 67 NIPPV = 66	81 (60.9)	69(51.9)	33 (30–35) ^a^	33 (30–37)^a^	1,880 (1,298–2693)^a^	1920 (1,405–2,968)^a^
3.	Zhu 2019 [37]	China, single	N = 103 NHFOV = 50 NIPPV = 53	45(43.7)	87(84.5)	29.7 ± 1.2	29.6 ± 1.4	1270 ± 115	1265 ± 120
4.	Jia 2021 [42]	China, single	N = 100 NHFOV = 50 NIPPV = 50	57 (57.0)	34(34)	31.89 ±1.42	31.77±1.50	1680 ± 350	1650±400
5.	Zhuang 2021 [29]	China, single	N = 90 NHFOV = 45 NIPPV = 45	56 (62.2)	29(32.2)	28.6±2.0	28.4±2.2	1 039±223	1029±230
6.	Liang 2019 [30]	China, single	N = 42 NHFOV = 21 NIPPV = 21	28(66.7)	NR	30.86±3.01	31.02±3.23	1472.34±102.55	1488.02±105.63
7.	Huang 2021 [43]	China, single	N = 130 NHFOV = 65 NIPPV = 65	67 (51.5)	84(64.6)	32. 95 ± 1. 65	32. 89 ± 1. 71	1728. 92 ±498. 78	1733. 33 ± 491. 65
8.	Zhang 2022 [28]	China, single	N = 41 NHFOV = 19 NIPPV = 22	27 (65.9)	NR	35.0 ± 1.8	34.2 ± 2.0	1.9 ± 0.3	1800 ± 0.2
9.	Ahmed 2023 [41]	Egypt, single	N = 60 NHFOV = 30 NIPPV = 30	36 (60.0)	43(71.7)	33.97±1.65	33.40±2.06	2280±0.52	2050±0.59

SD, standard deviation; NHFOV, non-invasive high frequency ventilation; NCPAP, nasal positive airway pressure; NIPPV, non-invasive positive pressure ventilation; NR, not reported.

^a^median (IQR)

**Table 3:** Characteristics of included studies (Studies comparing NHFOV vs NCPAP vs NIPPV).

	Study ID	Country & center	Sample size Total(N)			Gestational age(weeks), mean±SD			Birth weight (g), mean±SD		
				Male, n(%)	Antenatal steroids, n(%)	NHFOV	NCPAP	NIPPV	NHFOV	NCPAP	NIPPV
1.	Li 2021 [21]	China, single	N = 139 NHFOV = 45 NCPAP = 47 NIPPV = 47	130(93.5)	71(51.1)	29.1±1.9	29.0 ±1.7	28.9±2.0	1118.9±201.9	1132.1±202.5	1088.5±153.7
2.	Yuan 2022a(<32weeks) [26]	China, single	N = 120 NHFOV = 40 NCPAP = 40 NIPPV = 40	79(65.8)	60(50%)	30.60 ±1.71	30.12 ± 1.74	30.38 ± 1.61	1370 ± 0.33	1380 ± 0.28	1420 ± 0.30
	Yuan 2022b(32-36-6/7) [26]	China, single	N = 120 NHFOV = 40 NCPAP = 40 NIPPV = 40	73(60.8)	58(48.3)	33.86 ± 2.14	34.10 ± 2.15	33.90 ± 2.26	1990 ± 0.62	2120 ± 0.63	2030± 0.59
3.	Zhu 2022 [18]	China, single	N = 1440 NHFOV = 480 NCPAP = 480 NIPPV = 480	860 (59.7)	660 (45.8)	29·4±1·8	29.5±1.7	29.4 ±1.8	1317±353	1341±318	1334±366

SD, standard deviation; NHFOV, non-invasive high frequency ventilation; NCPAP, nasal positive airway pressure; NIPPV, non-invasive positive pressure ventilation.

### Study population

Among 21 included trials, 12 trials [18, 21, 23–26, 29, 31, 32, 37, 41, 42] included only preterm neonates and one trial [27] included only term neonates. One trial each included exclusively [21] newborns with meconium aspiration syndrome and persistent pulmonary hypertension of newborn (PPHN) [27], PPHN [28] and BPD [29], ten trials included exclusively neonates with RDS and the remaining trials included neonates with various conditions requiring NRS after extubation.

### Device and ventilator strategies

We found studies that compared NHFOV with NCPAP and NIPPV. Included studies used variable or continuous flow devices to deliver CPAP [18, 21, 24–26, 31, 32, 37]. Some studies did not mention the type of CPAP used [33–36]. Single trial exclusively compared synchronized NIPPV with NHFOV [20]. The largest trial by Zhu et al. [18] used only non-synchronized NIPPV. The information on synchronization was unavailable in other trials. Various ventilators were used to deliver NHFOV. Prespecified criteria for extubation failure were not reported in three trials [28, 42, 43]. Additionally, some studies did not mention the intervention carried out in case of NRS failure following extubation [27, 28, 33, 42, 43]. The definition of some secondary outcomes was not clear. The antenatal steroid coverage ranged from 32.2% to 93.5%.

### RoB

All included studies had high RoB for performance bias. Most studies had unclear RoB for allocation concealment, detection bias and reporting bias **(S1 and S2 Figs).** Details of RoB assessment are given in **S1 Appendix.**

### Effect of interventions

#### A. Comparison between NHFOV and NCPAP

**Fig 2:**
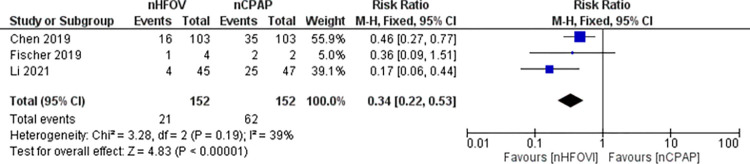
Forest plot of comparison: NHFOV vs NCPAP, outcome: Reintubation within 7 days of extubation.

*Primary outcome*. NHFOV reduced the incidence of reintubation within 7 days compared to NCPAP **(RR 0.34, 95% CI 0.22 to 0.53; participants = 304; studies = 3; I**^**2**^ **= 39%)** after extubation from mechanical ventilation **(Fig 2).** The NNT for successful treatment was 3.7 (95% CI 2.7 to 5.7).

**Fig 3:**
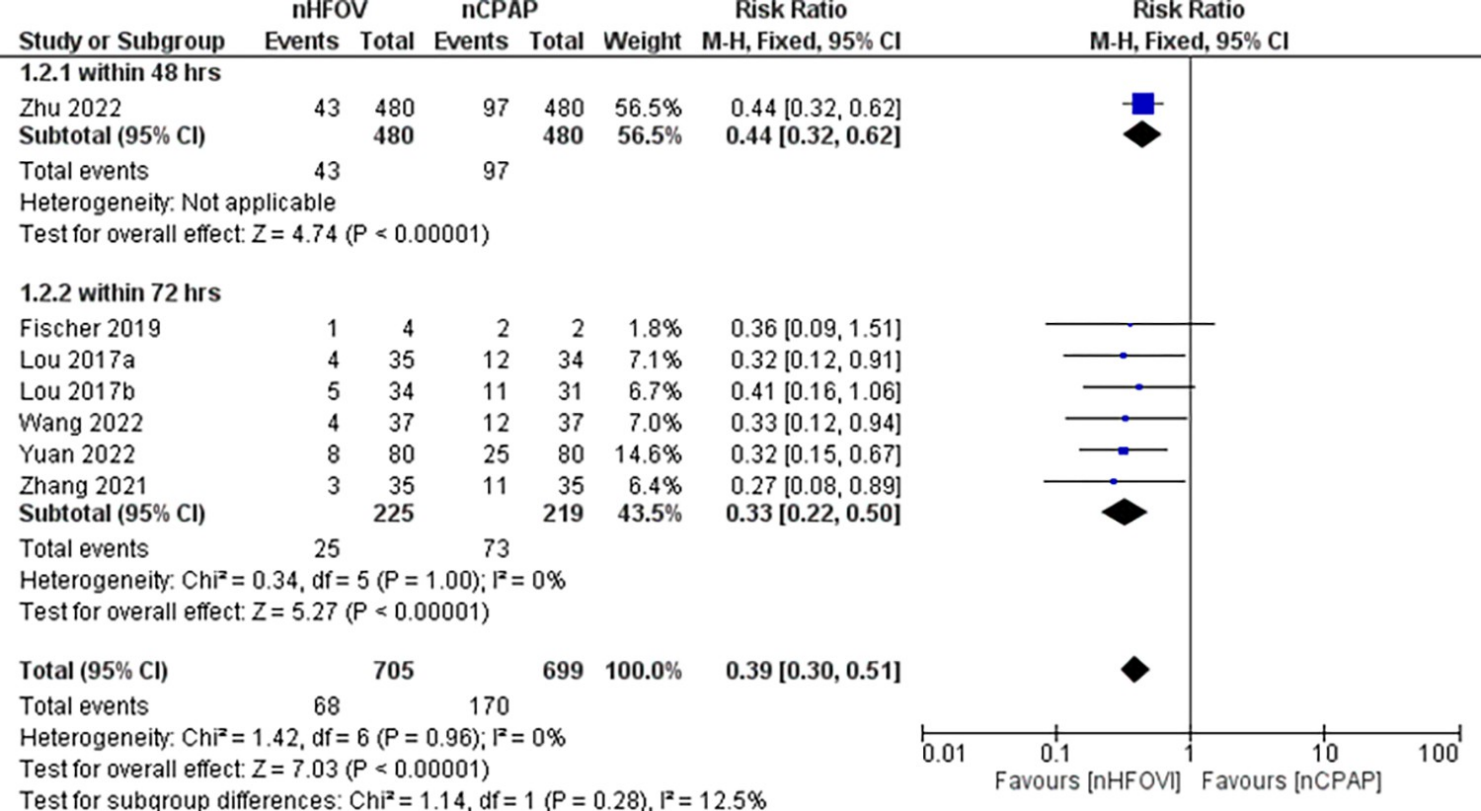
Forest plot of comparison: NHFOV vs NCPAP, outcome: Extubation failure within 72 hrs.

*Secondary outcomes*. All-trials analysis showed statistically significant reduction in extubation failure within 72 hours post-extubation **(RR 0.39, 95% CI 0.30 to 0.51; participants = 1404; studies = 7; I**^**2**^ **= 0%)** in neonates extubated to NHFOV **(Fig 3).** The NNT for successful treatment was 6.8(95% CI 5.4 to 9.2). Two studies [25, 43] reported extubation failure at 7 days and one of them reported data for both 72 hours and 7 days. Two RCTs that reported extubation failure at 7 days found no significant difference **(S3 Fig).**

One trial reported extubation failure rate but the trial did not specify the type of rescue treatment or intervention that was employed in the event of extubation failure [27]. One study reported extubation failure within 72 hrs and reintubation within 7 days [25]. All other trials employed reintubation after the failure of extubation within 72 hrs. A meta-analysis of RCTs that reported reintubation within 72 hrs (RR 0.40, 95% CI 0.31 to 0.53; participants = 1329; studies = 5; I^2^ = 0%) also found statistically significant reduction.

NHFOV resulted in lower rate of BPD (RR 0.59, 95% CI 0.37 to 0.94; participants = 1553; studies = 6; I^2^ = 57%), NNTB 14 (95% CI 8.6 to 37.5) **(S4A Fig),** and pulmonary air leak (RR 0.46, 95% CI 0.27 to 0.79; participants = 1896; studies = 11; I^2^ = 0%), NNTB 45 (95% CI 26.8 to 140.1) **(S4B Fig).** The rate of other secondary outcomes remained similar in both groups. The incidence of ROP of any grade **(RR 0.55, 95% CI 0.25 to 1.20; participants = 458; studies = 3; I**^**2**^ **= 52%) (S4C Fig),** ROP stage ≥3 (RR 0.85, 95% CI 0.62 to 1.16; participants = 960; studies = 1; I^2^ = 0%) **(S4D Fig),** and composite outcome of death/BPD (RR 0.90, 95% CI 0.77 to 1.06; participants = 966; studies = 2; I^2^ = 0%) **(S4E Fig)** were similar in both groups.

There was no difference in IVH any grade (RR 1.42, 95% CI 0.58 to 3.46; participants = 366; studies = 2; I^2^ = 61%) **(S5A Fig)** or IVH grade ≥3 ((RR 0.82, 95% CI 0.59 to 1.15; participants = 1187; studies = 4; I^2^ = 0%) **(S5B Fig),** all-cause mortality before hospital discharge (RR 0.99, 95% CI 0.48 to 2.06; participants = 1306; studies = 5; I^2^ = 0%) **(S5C Fig)** and nasal injury (RR 0.59, 95% CI 0.28 to 1.25; participants = 1487; studies = 5; I^2^ = 72%) **(S5D Fig)** in both groups. Studies that reported on IVH grade ≥3 and ROP, stage ≥3 were analysed separately from studies that reported IVH and ROP of any grade, respectively. LOS was reduced in NHFOV group (SMD -0.81, 95% CI -1.23 to -0.39; participants = 652; studies = 6; I^2^ = 84%) **(S5E Fig)**

#### B. Comparison between NHFOV and NIPPV

**Fig 4:**
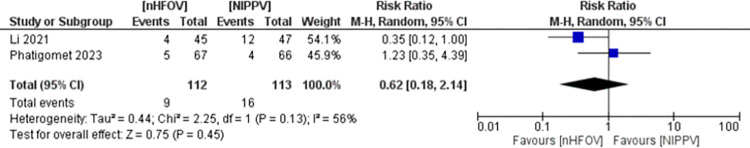
Forest plot of comparison: NHFOV vs NIPPV, outcome: Reintubation within 7 days of extubation.

*Primary outcome*. NHFOV did not reduce the incidence of reintubation within 7 days (RR 0.62, 95% CI 0.18 to 2.14; participants = 225; studies = 2; I^2^ = 56%) compared to NIPPV (**Fig 4)**.

**Fig 5:**
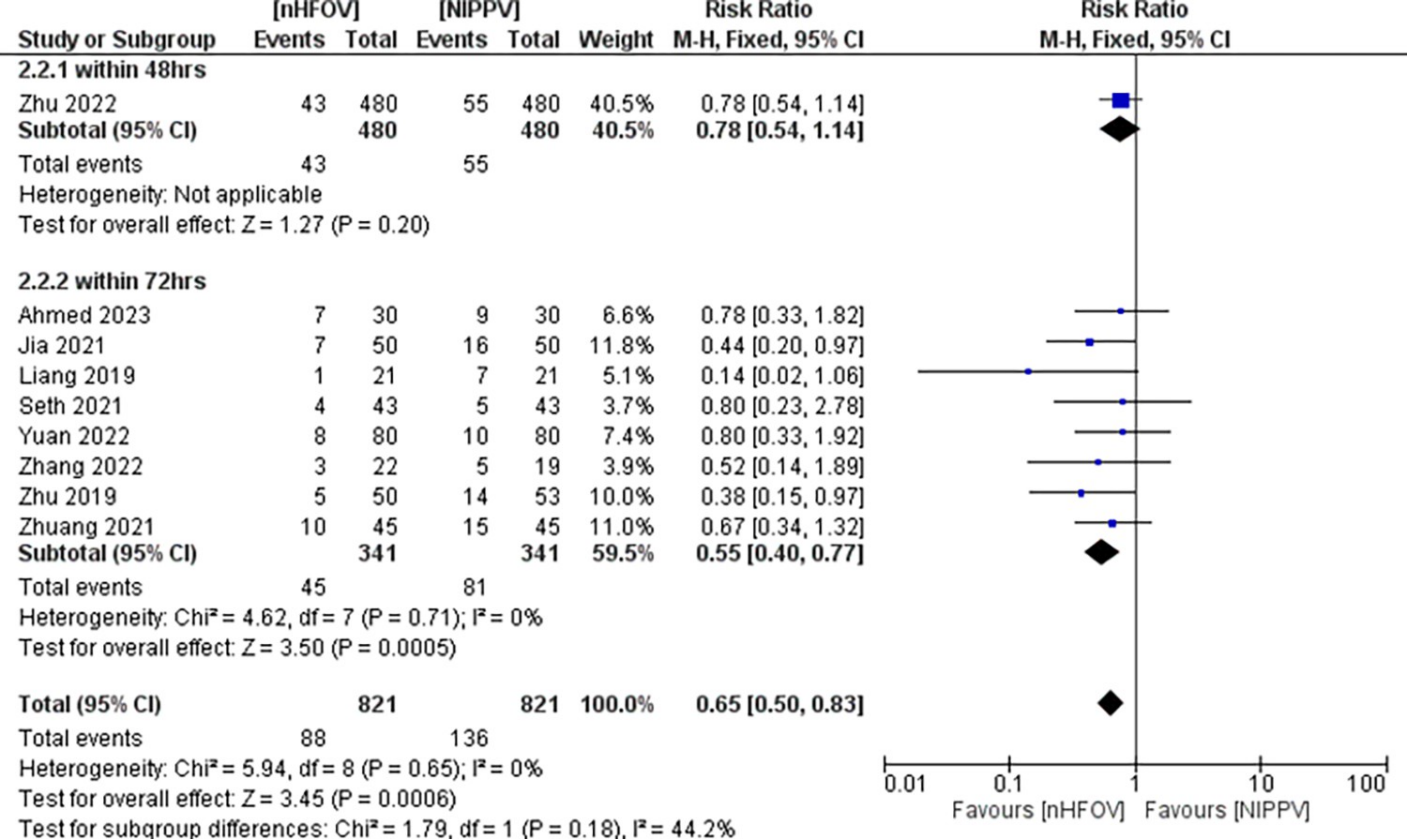
Forest plot of comparison: NHFOV vs NIPPV, outcome: Extubation failure within 72 hrs.

*Secondary outcomes*. NHFOV resulted in a reduction in the extubation failure rate within 72 hrs after extubation (RR 0.65, 95% CI 0.50 to 0.83; participants = 1642; studies = 9; I^2^ = 0%), NNTB 17.1 (95% CI 10.9 to 39.4) **(Fig 5)**. Two studies [28, 42] reported the extubation failure rate. Neither of these two studies provided any pre-specified extubation failure criteria or described interventions in the event of failure. All other studies reported reintubation. After removing these two studies, reintubation rate within 72 hrs remained statistically significant (RR 0.68, 95% CI 0.52 to 0.89).

There was no difference in other secondary outcomes between NHFOV and NIPPV. The risk of BPD (RR 0.88, 95% CI 0.76 to 1.02; participants = 1659; studies = 8; I^2^ = 0%) **(S6A Fig)**, pulmonary air leak (RR 0.82, 95% CI 0.43 to 1.53; participants = 1694; studies = 8; I^2^ = 5%) **(S6B Fig)**, ROP of any stage (RR 0.79, 95% CI 0.47 to 1.32; participants = 507; studies = 5; I^2^ = 0%) **(S6C Fig)**, ROP stage ≥3 (RR 0.87, 95% CI 0.64 to 1.19; participants = 1029; studies = 2; I^2^ = 0%) **(S6D Fig)** and composite outcome of death/BPD (RR 0.91, 95% CI 0.78 to 1.07; participants = 1046; studies = 2; I^2^ = 0%) **(S6E Fig)** were similar in both groups.

There was no difference in IVH any stage (RR 1.43, 95% CI 0.41 to 4.95; participants = 305; studies = 3; I^2^ = 86%) **(S7A Fig)**, and IVH stage ≥3 (RR 0.80, 95% CI 0.57 to 1.12; participants = 1326; studies = 5; I^2^ = 0%) **(S7B Fig).** all-cause mortality before hospital discharge (RR 0.98, 95% CI 0.25 to 3.84; participants = 1179; studies = 3; I^2^ = 64%) **(S7C Fig),** and nasal injury (RR 1.08, 95% CI 0.89 to 1.31; participants = 1517; studies = 7; I^2^ = 0%) **(S7D Fig)**, between NHFOV and NIPPV group. LOS was similar in both groups (SMD -0.06, 95% CI -0.23 to 0.11; participants = 561; studies = 5; I^2^ = 0%) **(S7E Fig).** Seth et al. [23] provided additional data on BPD, mortality at discharge, LOS, ROP of any stage and IVH ≥grade 3 which were pre-specified in their protocol.

### Quality of the evidence

The overall quality of evidence as per GRADE is provided in the Summary of Findings tables **(S3 and S4 Tables).** Lack of blinding to outcome assessor was not considered a source of bias for mortality, BPD, pulmonary air leak, ROP stage ≥3 and IVH grade ≥3. We assessed the Certainty of evidence as moderate for pulmonary air leak and low to very low for other outcomes in comparison between NHFOV and NCPAP. The certainty of evidence was low to very low for all outcomes in comparison between NHFOV and NIPPV.

### Subgroup analysis and sensitivity analysis

#### Meta-analysis restricted to preterm neonates

Separate meta-analyses for other outcomes by restricting the analyses to preterm neonates (<37 weeks gestation) revealed no change in the pattern of results for both comparisons **(S8 and S9 Figs).** The effect estimates are summarized in **S5** and **S6 Tables.**

#### Comparison of NHFOV with NCPAP and NIPPV in term neonates

Meta-analysis including only term neonates was not possible for any of the outcomes due to insufficient data. Only one study [27] exclusively included term neonates. Lou et al. [27] reported reduced extubation failure rate (11.43%, vs 35.29%, P<0.05) and nasal injury (5.71% vs 32.35%) in NHFOV group compared to NCPAP. The rate of pulmonary air leak was similar in both groups.

Phatigomet et al. reported reintubation rate within 7 days in 8.33% (1/12) and 4.55% (1/22) term neonates in NHFOV and SNIPPV group respectively [20].

#### Subgroup analysis for RoB

For sensitivity analysis, we divided studies into two subgroups on the basis of RoB: (i) low-risk-of-bias trials- studies that had low or unclear risk of bias only in blinding of outcome assessors (in addition to high risk of bias in blinding of participants and personnel). (ii) high-risk-of-bias trials- studies that, in addition, had unclear or high risk of bias in one or more of the other domains for risk of bias. Subgroup analysis was not possible for all outcomes. The overall results did not change significantly in low-risk-of-bias trials for any outcome except for pulmonary air leak (NHFOV vs NCPAP) and extubation failure (NHFOV vs NIPPV).

*NHFOV vs NCPAP*. Low-risk-of-bias trials showed no difference whereas high-risk-of-bias subgroup showed a significant difference in the incidence of pulmonary air leak in favour of NHFOV **(S7 Table).**

*NHFOV vs NIPPV*. Low-risk-of-bias trials showed no reduction whereas high-risk-of-bias trials showed a significant reduction in extubation failure rate in NHFOV group **(S8 Table).**

#### Addressing heterogeneity and leave-one-out meta-analysis

*NHFOV vs NCPAP*. There was unexplained moderate to high heterogeneity in BPD, nasal injury and LOS. We drew Baujat plot which detected influential studies contributing to heterogeneity **(S10 Fig).** Leave-one-out meta-analysis revealed substantial reduction in heterogeneity and change in effect estimate after removing influential studies **(S11 Fig).** Additionally, subgrouping by preterm and low-risk-of-bias trials eliminated heterogeneity in LOS (I^2^ = 0).

*NHFOV vs NIPPV*. Moderate heterogeneity was observed in reintubation within seven days and mortality before hospital discharge which may be explained by clinical heterogeneity.

Finally, we ran a "leave-one-out" sensitivity analysis, omitting one study at a time, to see if the findings were influenced by a single study. Leave-one-out meta-analysis revealed that the effect estimates for extubation failure remained stable in both comparisons **(S12 Fig).** Some of the effect estimates substantially changed reflecting influential studies.

#### Publication bias

Visual inspection of funnel plot revealed asymmetry for LOS in comparison between NHFOV and NCPAP and for nasal injury in comparison between NHFOV and NIPPV **(S13 Fig).** There was no evidence of funnel plot asymmetry for other outcomes in both comparisons.

## Discussion

In this meta-analysis, we found that NHFOV reduced the incidence of reintubation at seven days compared to NCPAP but not NIPPV. However, extubation failure and reintubation within 72 hours were lower in the NHFOV group compared to both NCPAP and NIPPV. We found lower risk of BPD, pulmonary air leak and ROP of any stage and shorter LOS in NHFOV group compared to NCPAP. There was a trend towards lower BPD when NHFOV was compared to NIPPV. However, NHFOV did not significantly impact other outcomes. The estimates of effects were similar when including only preterm neonates. The overall quality of evidence was low to very low.

We conducted subgroup analysis to determine if the methodological quality of the included trials influenced the treatment effect estimates. In the subgroup of studies with low risk of bias, the effect estimates remained statistically significant for extubation failure within 72 hours, BPD and LOS and non-significant for mortality, nasal injury, and IVH grade ≥3 when comparing NHFOV and NCPAP. In comparison between NHFOV vs NIPPV, low-risk-of-bias trials showed no difference whereas high-risk-of-bias trials showed reduction in extubation failure. The results of other outcomes did not show significant change in RoB subgroup analysis. This suggests that the results for these outcomes were robust and consistent. Furthermore, we found that high-risk-of-bias trials overestimated the effect estimates for some secondary outcomes. Sufficient data were available only for preterm neonates to do subgroup meta-analysis. A significant proportion of the participants in the included studies were preterm neonates. As a result, the trial level subgroup analysis focusing on preterm neonates have not yielded a dramatic change in effect estimates. Leave one out meta-analysis revealed that some of the results were sensitive for removal of studies. Meta-analyses enhance statistical power and precision in determining the studied effect. However, excluding a single study is likely to alter the pooled effect estimate, sometimes in a significant manner particularly if there are only a few numbers of studies.

An association between reintubation within 72 hrs of extubation and increased likelihood of moderate-to-severe BPD or death in very low birth weight infants was reported [44] Here, we found that NHFOV probably reduces the risk of extubation failure or reintubation within 72 hrs in NICU patients when compared to NCPAP or NIPPV. The decreased rate of reintubation probably played a role in the lower occurrence of BPD in neonates receiving NHFOV. In addition to its effectiveness, our review demonstrated NHFOV’s safety for newborns.

### Strength

This review has multiple strengths such as an extensive search and a pre-registered protocol. We included 21 RCTs with a total of 3294 newborns. We included the most recently published RCTs and articles published in non-English language in our review. It incorporates substantial new data thus contributing to increased precision in estimating treatment effects. We analysed extubation failure and reintubation as distinct outcomes. Previous reviews focussed only on preterm neonates whereas we studied a broad research question and included neonates of all gestational ages. This allowed us to identify several RCTs which included term neonates or mixed population. We conducted subgroup and sensitivity analysis to determine the robustness of the results and explored heterogeneity. Our exploratory review highlights several evidence gaps for future research. We contacted authors of registered clinical trials that met our inclusion criteria to find additional results to include in this review. Furthermore, trials with methodologically good quality contributed to the majority of the data to the meta-analyses.

### Limitations

Most of the included RCTs were conducted in developing countries, with a notable paucity of studies from developed countries. Most trials were small, single-centre studies and published from low- and middle-income countries. Caregivers cannot be blinded due to the nature of the interventions which may have led to bias. Majority of the population was preterm in our meta-analysis. We were unable to do meaningful subgroup analysis for full term, very preterm, and extremely preterm neonates due to limited data. There was limited information on the type of NCPAP or synchronization in NIPPV. Some studies lacked defined criteria for extubation failure and some secondary outcomes, and a few didn’t specify interventions following NRS failure after extubation. No study reported mortality at 28 days of life. We didn’t find any study that compared NHFOV with NIV-NAVA, HFNC or oxygen/no treatment.

The incidence of BPD was relatively high among neonates, regardless of the interventions studied. The antenatal steroid coverage was notably poor in majority of the trials. These specific characteristics could have impacted our findings. Furthermore, factors like equipment availability, costs to patients and the healthcare system, and the level of nursing care vary across health systems and regions that may potentially impact the efficacy of different NRS modes. Thus, the results might differ in populations with different characteristics or in settings with different expertise. Additionally, diverse ventilator settings and extubation failure criteria might also affect the efficacy of NRS.

Previous reviews also found reduction in reintubation rate when NHFOV was compared to NIPPV although these reviews included only preterm and fewer articles [9, 10]. Similarly, previous reviews did not show any effect on other outcomes when NHFOV was compared with NIPPV [9, 10]. A network meta-analysis showed reduction in reintubation rate within seven days of extubation with NHFOV compared to continuous flow CPAP but not when compared to variable flow CPAP or NIPPV [8]. The largest trial by Zhu et al. [18] similarly showed a decrease in early reintubation in NHFOV group as compared to NCPAP groups. However, compared to NIPPV, this reduction did not achieve statistical significance.

Extubation failure has both prognostic and patient-related importance. Interventions that can reduce reintubation risk are valuable to a wide range of stakeholders involved in patient care and decision-making. The majority of the participants included in this review received non-synchronized NIPPV. As SNIPPV might result in better outcome, this mode needs to be compared with NHFOV in future trials. Future trials should include well-defined homogenous populations. Our results are mainly applicable to preterm neonates. There is paucity of research in term neonates and in conditions like BPD and MAS. Different nasal interfaces can impact the delivery of NHFOV and affect patient outcomes. Multi-center trials from developed countries with rigorous methodology are needed to further confirm the efficacy and safety of NHFOV.

## Conclusion

Despite some limitations, in this meta-analysis NHFOV demonstrated reduction in reintubation following extubation when compared to NCPAP or NIPPV. Importantly, the use of NHFOV did not appear to increase the risk of serious complications. However, caution is warranted in interpreting the results due to the low to very low certainty of evidence. Further multi-center randomized controlled trials are warranted to confirm the effectiveness and safety of NHFOV as post-extubation respiratory support.

## Supporting information

S1 Checklist PRISMA 2020 for abstract checklist. (DOCX)

S2 Checklist PRISMA 2020 checklist. (DOCX)

S1 Fig Risk of bias graph: Review authors’ judgements about each risk of bias item presented as percentages across all included studies. (TIF)

S2 Fig Risk of bias summary: Review authors’ judgements about each risk of bias item for each included study. (TIF)

S3 Fig Forest plot of comparison: NHFOV vs NCPAP, outcome: Extubation failure at 7 days. (TIF)

S4 Fig Forest plot of comparison: NHFOV vs NCPAP, outcome: (A) Bronchopulmonary dysplasia; (B) Pulmonary air leak; (C) Retinopathy of prematurity, any stage; (D) Retinopathy of prematurity, severe stage ≥3; (E) Composite outcome of death/BPD.
(TIF)

S5 Fig Forest plot of comparison: NHFOV vs NCPAP, outcome: (A) Intraventricular haemorrhage, any grade; (B) Intraventricular haemorrhage, grade ⪰3; (C) All-cause mortality (before hospital discharge); (D) Nasal injury; (E) Length of hospital stay, days.
(TIF)

S6 Fig Forest plot of comparison: NHFOV vs NIPPV, outcome: (A) Bronchopulmonary dysplasia; (B) Pulmonary air leak; (C) Retinopathy of prematurity, any stage; (D) Retinopathy of prematurity, severe stage ≥3; (E) Composite outcome of death/BPD.
(TIF)

S7 Fig Forest plot of comparison: NHFOV vs NIPPV, outcome: (A) Intraventricular haemorrhage, any grade; (B) Intraventricular haemorrhage, grade ⪰3; (C) All-cause mortality (before hospital discharge); (D) Nasal injury; (E) Length of hospital stay, days.
(TIF)

S8 Fig Forest plot of comparison: NHFOV vs NCPAP (preterm subgroup), outcome: (A) Extubation failure; (B) Pulmonary air leak; (C) All-cause mortality (before hospital discharge); (D) Nasal injury; (E) Length of hospital stay, days.
(TIF)

S9 Fig Forest plot of comparison: NHFOV vs NIPPV (preterm subgroup), outcome: (A) Reintubation (within 7 days of extubation); (B) Extubation failure; (C) Pulmonary air leak; (D) All-cause mortality (before hospital discharge); (E) Nasal injury; (F) Length of hospital stay, days.
(TIF)

S10 Fig Baujat plot showing the studies that contributed to heterogeneity of comparison NHFOV vs NCPAP, outcome: (A) Bronchopulmonary dysplasia; (B) Nasal injury; (C) Length of hospital stay, days.
(TIF)

S11 Fig Leave-one-out meta-analysis: NHFOV vs NCPAP, outcome: (A) Bronchopulmonary dysplasia; (B) Nasal injury; (C) Length of hospital stay.
(TIF)

S12 Fig Leave-one-out meta-analysis of Extubation failure, comparison: (A) NHFOV vs NCPAP; (B) NHFOV vs NIPPV.
(TIF)

S13 Fig Funnel plot showing publication bias: (A) comparison NHFOV vs NCPAP, outcome: Length of hospital stay; (B) comparison NHFOV vs NIPPV, outcome: Nasal injury.
(TIF)

S1 Table Search strategy. (DOCX)

S2 Table List of excluded studies. (DOCX)

S3 Table NHFOV compared to NCPAP for respiratory support after extubation in neonates. (DOCX)

S4 Table NHFOV compared to NIPPV for respiratory support after extubation in neonates. (DOCX)

S5 Table NHFOV versus NCPAP: Subgroup analysis for preterm. (ODT)

S6 Table NHFOV versus NIPPV: Subgroup analysis for preterm neonates. (ODT)

S7 Table NHFOV vs NCPAP, subgroup analysis for risk of bias. (ODT)

S8 Table NHFOV vs NIPPV, subgroup analysis for risk of bias. (ODT)

S1 Appendix Data extraction and assessment of methodological quality. (DOCX)

## References

[1] Jensen EA, DeMauro SB, Kornhauser M, Aghai ZH, Greenspan JS, Dysart KC. Effects of multiple ventilation courses and duration of mechanical ventilation on respiratory outcomes in extremely low-birth-weight infants. JAMA pediatrics. 2015;169(11):1011–7. doi: 10.1001/jamapediatrics.2015.2401 26414549 PMC6445387

[2] Lemyre B, Deguise MO, Benson P, Kirpalani H, De Paoli AG, Davis PG. Nasal intermittent positive pressure ventilation (NIPPV) versus nasal continuous positive airway pressure (NCPAP) for preterm neonates after extubation. Cochrane Database of Systematic Reviews. 2023;(7). doi: 10.1002/14651858.CD003212.pub4 CD003212. 37497794 PMC10374244

[3] Bottino R, Pontiggia F, Ricci C, Gambacorta A, Paladini A, Chijenas V, et al. Nasal high-frequency oscillatory ventilation and CO(2) removal: A randomized controlled crossover trial. Pediatr Pulmonol. 2018;53(9):1245–51. Epub 20180712. doi: 10.1002/ppul.24120 . 29999596

[4] Li J, Li X, Huang X, Zhang Z. Noninvasive high-frequency oscillatory ventilation as respiratory support in preterm infants: a meta-analysis of randomized controlled trials. Respiratory Research. 2019;20(1):58. doi: 10.1186/s12931-019-1023-0 30876411 PMC6420773

[5] Fischer HS, Bohlin K, Bührer C, Schmalisch G, Cremer M, Reiss I, et al. Nasal high-frequency oscillation ventilation in neonates: a survey in five European countries. European Journal of Pediatrics. 2015;174(4):465–71. doi: 10.1007/s00431-014-2419-y 25227281

[6] Li J, Chen L, Shi Y. Nasal high-frequency oscillatory ventilation versus nasal continuous positive airway pressure as primary respiratory support strategies for respiratory distress syndrome in preterm infants: a systematic review and meta-analysis. Eur J Pediatr. 2022;181(1):215–23. Epub 20210712. doi: 10.1007/s00431-021-04190-0 . 34254173

[7] De Luca D, Centorrino R. Nasal High-Frequency Ventilation. Clin Perinatol. 2021;48(4):761–82. Epub 20211002. doi: 10.1016/j.clp.2021.07.006 . 34774208

[8] Ramaswamy VV, Bandyopadhyay T, Nanda D, Bandiya P, More K, Oommen VI, et al. Efficacy of noninvasive respiratory support modes as postextubation respiratory support in preterm neonates: A systematic review and network meta‐analysis. Pediatric pulmonology. 2020;55(11):2924–39. doi: 10.1002/ppul.25007 32757365

[9] Mei Z, Ming L, Wu Z, Zhu Y. Use of NHFOV vs. NIPPV for the respiratory support of preterm newborns after extubation: A meta-analysis. Front Pediatr. 2022;10:1063387. Epub 20230111. doi: 10.3389/fped.2022.1063387 ; PubMed Central PMCID: PMC9874940. 36714640 PMC9874940

[10] Wang K, Zhou X, Gao S, Li F, Ju R. Noninvasive high-frequency oscillatory ventilation versus nasal intermittent positive pressure ventilation for preterm infants as an extubation support: A systematic review and meta-analysis. Pediatr Pulmonol. 2023;58(3):704–11. Epub 20221228. doi: 10.1002/ppul.26244 . 36372443

[11] Page MJ, McKenzie JE, Bossuyt PM, Boutron I, Hoffmann TC, Mulrow CD, et al. The PRISMA 2020 statement: an updated guideline for reporting systematic reviews. BMJ. 2021;372:n71. doi: 10.1136/bmj.n71 33782057 PMC8005924

[12] Papile LA, Burstein J, Burstein R, Koffler H. Incidence and evolution of subependymal and intraventricular hemorrhage: a study of infants with birth weights less than 1,500 gm. J Pediatr. 1978;92(4):529–34. doi: 10.1016/s0022-3476(78)80282-0 305471

[13] Chiang MF, Quinn GE, Fielder AR, Ostmo SR, Paul Chan RV, Berrocal A, et al. International Classification of Retinopathy of Prematurity, Third Edition. Ophthalmology. 2021;128(10):e51–e68. Epub 20210708. doi: 10.1016/j.ophtha.2021.05.031 . 34247850 PMC10979521

[14] Higgins JPT, Altman DG, Gøtzsche PC, Jüni P, Moher D, Oxman AD, et al. The Cochrane Collaboration’s tool for assessing risk of bias in randomised trials. BMJ. 2011;343:d5928. doi: 10.1136/bmj.d5928 22008217 PMC3196245

[15] Schünemann H, Brożek J, Guyatt G, Oxman A. GRADE handbook for grading quality of evidence and strength of recommendations. The GRADE Working Group; 2013. Available from: guidelinedevelopment org/handbook. 2018.

[16] Wan X, Wang W, Liu J, Tong T. Estimating the sample mean and standard deviation from the sample size, median, range and/or interquartile range. BMC Medical Research Methodology. 2014;14(1):135. doi: 10.1186/1471-2288-14-135 25524443 PMC4383202

[17] Shi Y, De Luca D, group NAOp-Es. Continuous positive airway pressure (CPAP) vs noninvasive positive pressure ventilation (NIPPV) vs noninvasive high frequency oscillation ventilation (NHFOV) as post-extubation support in preterm neonates: protocol for an assessor-blinded, multicenter, randomized controlled trial. BMC Pediatr. 2019;19(1):256. Epub 20190726. doi: 10.1186/s12887-019-1625-1 ; PubMed Central PMCID: PMC6659219. 31349833 PMC6659219

[18] Zhu X, Qi H, Feng Z, Shi Y, De Luca D, Nasal Oscillation Post-Extubation Study G. Noninvasive High-Frequency Oscillatory Ventilation vs Nasal Continuous Positive Airway Pressure vs Nasal Intermittent Positive Pressure Ventilation as Postextubation Support for Preterm Neonates in China: A Randomized Clinical Trial. JAMA Pediatr. 2022;176(6):551–9. doi: 10.1001/jamapediatrics.2022.0710 ; PubMed Central PMCID: PMC9039831. 35467744 PMC9039831

[19] Zhu X, Li F, Shi Y, Feng Z, De Luca D, Nasal Oscillation Post-Extubation Study G. Effectiveness of Nasal Continuous Positive Airway Pressure vs Nasal Intermittent Positive Pressure Ventilation vs Noninvasive High-Frequency Oscillatory Ventilation as Support After Extubation of Neonates Born Extremely Preterm or With More Severe Respiratory Failure: A Secondary Analysis of a Randomized Clinical Trial. JAMA Netw Open. 2023;6(7):e2321644. Epub 20230703. doi: 10.1001/jamanetworkopen.2023.21644 ; PubMed Central PMCID: PMC10318479. 37399009 PMC10318479

[20] Phatigomet M, Thatrimontrichai A, Maneenil G, Dissaneevate S, Janjindamai W. Reintubation Rate between Nasal High-Frequency Oscillatory Ventilation versus Synchronized Nasal Intermittent Positive Pressure Ventilation in Neonates: a Parallel Randomized Controlled Trial. Am J Perinatol. 2023. Epub 20230627. doi: 10.1055/a-2118-5351 . 37369239

[21] Li Y, Wei Q, Zhao D, Mo Y, Yao L, Li L, et al. Non-invasive high-frequency oscillatory ventilation in preterm infants after extubation: a randomized, controlled trial. J Int Med Res. 2021;49(2):300060520984915. doi: 10.1177/0300060520984915 ; PubMed Central PMCID: PMC7923990. 33641473 PMC7923990

[22] Li Y, Mo Y, Yao L, Wei Q, Meng D, Tan W, et al. The long-term outcomes of preterm infants receiving non-invasive high-frequency oscillatory ventilation. Front Pediatr. 2022;10:865057. Epub 20220722. doi: 10.3389/fped.2022.865057 ; PubMed Central PMCID: PMC9353142. 35935354 PMC9353142

[23] Seth S, Saha B, Saha AK, Mukherjee S, Hazra A. Nasal HFOV versus nasal IPPV as a post-extubation respiratory support in preterm infants-a randomised controlled trial. Eur J Pediatr. 2021;180(10):3151–60. Epub 20210423. doi: 10.1007/s00431-021-04084-1 ; PubMed Central PMCID: PMC8062142. 33890156 PMC8062142

[24] Chen L, Wang L, Ma J, Feng Z, Li J, Shi Y. Nasal High-Frequency Oscillatory Ventilation in Preterm Infants With Respiratory Distress Syndrome and ARDS After Extubation: A Randomized Controlled Trial. Chest. 2019;155(4):740–8. doi: 10.1016/j.chest.2019.01.014 . 30955572

[25] Fischer HS, Buhrer C, Czernik C. Hazards to avoid in future neonatal studies of nasal high-frequency oscillatory ventilation: lessons from an early terminated trial. BMC Res Notes. 2019;12(1):237. Epub 20190425. doi: 10.1186/s13104-019-4268-2 ; PubMed Central PMCID: PMC6482494. 31023363 PMC6482494

[26] Yuan G, Liu H, Wu Z, Chen X. Evaluation of three non-invasive ventilation modes after extubation in the treatment of preterm infants with severe respiratory distress syndrome. J Perinatol. 2022;42(9):1238–43. Epub 20220811. doi: 10.1038/s41372-022-01461-y . 35953535

[27] Lou W, Weixing Z, Li Y, Bing Z. Application of noninvasive high frequency oscillatory ventilation in neonates with meconium aspiration syndrome with pulmonary hypertension after extubation. The Journal of Practical Medicine. 2017;33(23):3919–23.

[28] Zhang R, Shen L, Wen J, Ye L, Liang Z, Han F. Application of Non-invasive High Frequency Oscillation Ventilation after Extubation in Persistent Pulmonary Hypertension of Newborn. Shenzhen Journal of Integrated Traditional Chinese and Western Medicine. 2022;32(01):101–3. doi: 10.16458/j.cnki.1007-0893.2022.01.032

[29] Zhuang Y, Gao X, Wu Y, Xiong Y, Zhou D. Application of noninvasive high-frequency oscillatory ventilation after extubation in preterm infants with severe bronchopulmonary dysplasia. Chin J Neonatol. 2021;36(02):42–7. doi: 10.3760/cma.j.issn.2096-2932.2021.02.008

[30] Liang Z, Chen N, Wang W. Evaluation on effects of non-invasive high-frequency ventilation on respiratory support for neonatal acute respiratory distress syndrome after removal of ventilator. China Medicine and Pharmacy. 2019;9(18):110–2.

[31] Lou W, Zhang W. Noninvasive high-frequency oscillatory ventilation versus nasal continuous positive airway pressure in premature infants with respiratory distress syndrome after weaning: A randomized controlled trial. Guangdong Medical Journal. 2017;38(13):2037–40.

[32] Zhang H, Fu H. Non-invasive High Frequency Oscillatory Ventilation Versus Nasal Continuous Positive Airway Pressure as Postextubation Respiratory Support in Preterm Neonates. Journal of Hubei University of Medicine. 2021;40(04):391–5.

[33] Cao M, Wang Y, Lan Q. Application Value of Noninvasive High Frequency Ventilation in the Weaning Process of Neonatal Respiratory Distress Syndrome. Chinese and Foreign Medical Research. 2019;17(15):14–5.

[34] Wang W. Evaluation of the effect of noninvasive high frequency ventilation on respiratory support after weaning from mechanical ventilation of neonatal acute respiratory distress syndrome. Chinese Communuty Doctors. 2020;36(25):35–6.

[35] Wang W. Study on Non-invasive High Frequency Ventilation Combined with Caffeine in Neonatal Respiratory Distress Syndrome Withdrawal. China &Foreign Medical Treatment. 2022;41(31):123–6+35.

[36] Guo Z, Chen L, Tao L, Zhou M. Clinical study of NHFOV on preventing extubation. China Modern Medicine. 2021;28(23):127–30.

[37] Wang Z, Gao Ww, Shen Y, Lin X, Shen Yz, Zhou Wj, et al. Effects of noninvasive high frequency oscillatory ventilation for respiratory distress syndrome in very low birth weight preterm infants after extubation. Guangdong Medical Journal. 2019;40(10):1391–5.

[38] Mukerji A, Sarmiento K, Lee B, Hassall K, Shah V. Non-invasive high-frequency ventilation versus bi-phasic continuous positive airway pressure (BP-CPAP) following CPAP failure in infants <1250 g: a pilot randomized controlled trial. J Perinatol. 2017;37(1):49–53. Epub 20160929. doi: 10.1038/jp.2016.172 . 27684415

[39] Li H, Zhu X, Wang W. Efficacy of non-invasive high frequency oscillatory ventilation as post-extubation respiratory support in preterm neonates: a randomized controlled trial. Journal of Third Military Medical University. 2019;41(17):1688–92.

[40] Lou W, Zhang W-X, Yuan L, Zhang B. Comparative Study of Noninvasive High-frequency Oscillatory Ventilation and Bilevel Positive Airway Pressure Ventilation for Preterm Infants with Respiratory Distress Syndrome. Chinese General Practice. 2018;21(16):1983–8.

[41] Ahmed WO, AbuSaif ISH, Salaheldin SA, Hashem HE, Obaid OA, Obaid AA, et al. Noninvasive high frequency oscillatory ventilation versus noninvasive positive pressure ventilation in preterm neonates after extubation: A randomized controlled trial. Journal of Neonatal-Perinatal Medicine. 2023;Preprint:1–10. doi: 10.3233/NPM-221199 37718865

[42] Jia Y, Zhao G, Zhang K. Effect of different ventilation modes on low birth weight premature infants with respiratory distress syndrome after extubation and weaning. Chinese Journal of Practical Medicine. 2021;48(12):49–52.

[43] Huang X, Liu Y, Zhuang F. Comparison of the effect of non-invasive high frequency ventilation and nasal intermittent positive pressure ventilation in the treatment of neonatal respiratory distress syndrome after withdrawal. Clinical Medicine. 2021;41(06):69–70. doi: 10.19528/j.issn.1003-3548.2021.06.026

[44] Li J, Zhang J, Hao Q, Shen Z, Du Y, Chen H, et al. The Impact of Time Interval Between First Extubation and Reintubation on Bronchopulmonary Dysplasia or Death in Very Low Birth Weight Infants. Front Pediatr. 2022;10:867767. Epub 20220425. doi: 10.3389/fped.2022.867767 ; PubMed Central PMCID: PMC9085302. 35547548 PMC9085302

