# Supplementary figures and images for: Noninvasive high-frequency oscillation ventilation as post- extubation respiratory support in neonates: Systematic review and meta-analysis
Source: PLoS One. 2024 Jul 30;19(7):e0307903. doi: 10.1371/journal.pone.0307903 (PMC11288463; doi:10.1371/journal.pone.0307903)

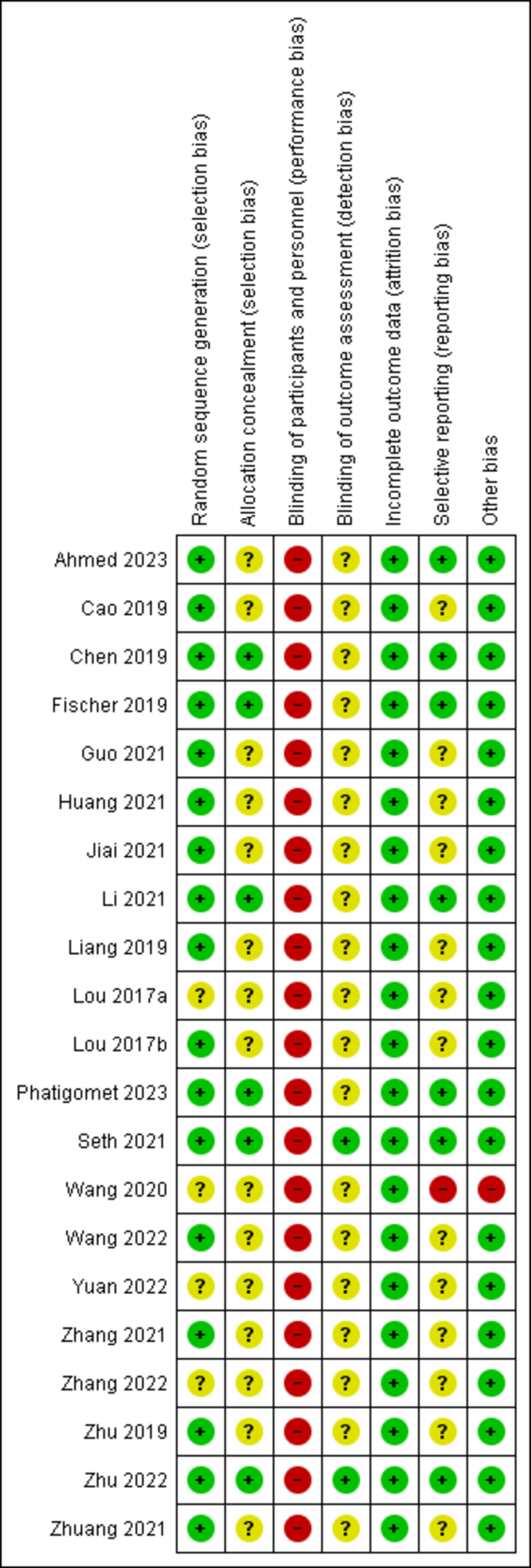

Supplement: S1 Fig — (TIF) [file pone.0307903.s003.tif]

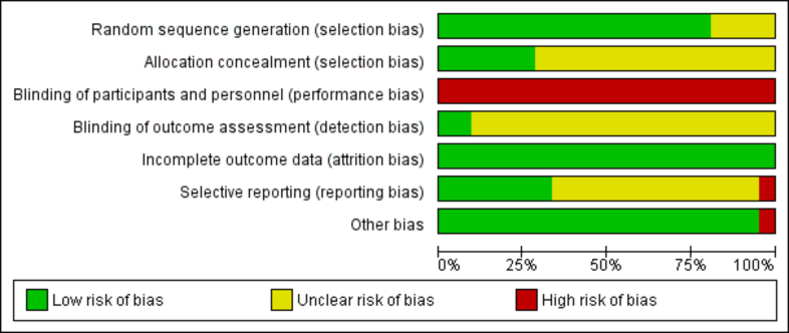

Supplement: S2 Fig — (TIF) [file pone.0307903.s004.tif]

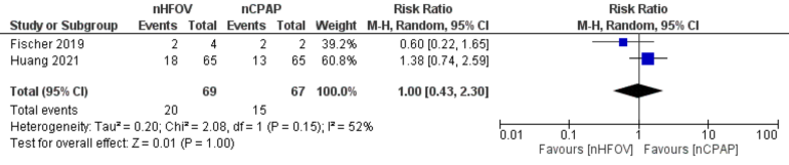

Supplement: S3 Fig — (TIF) [file pone.0307903.s005.tif]

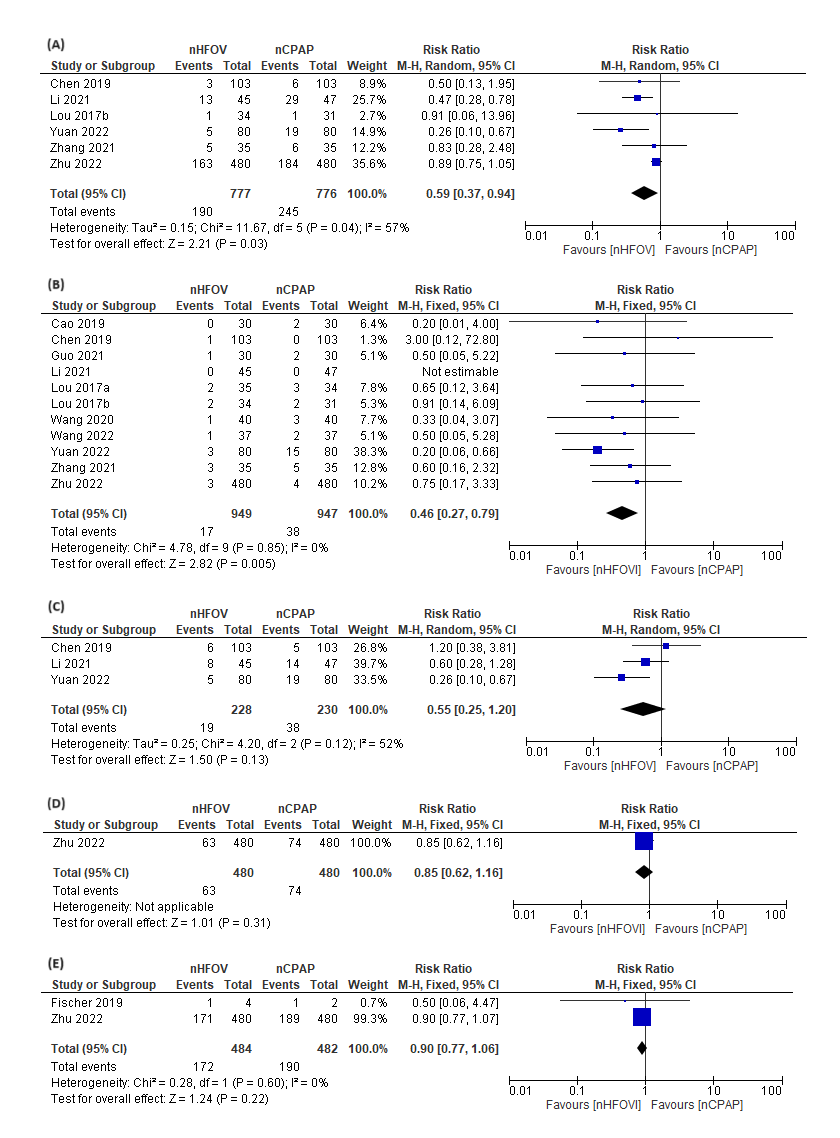

Supplement: S4 Fig — Forest plot of comparison: NHFOV vs NCPAP, outcome: (A) Bronchopulmonary dysplasia; (B) Pulmonary air leak; (C) Retinopathy of prematurity, any stage; (D) Retinopathy of prematurity, severe stage ≥3; (E) Composite outcome of death/BPD. (TIF) [file pone.0307903.s006.tif]

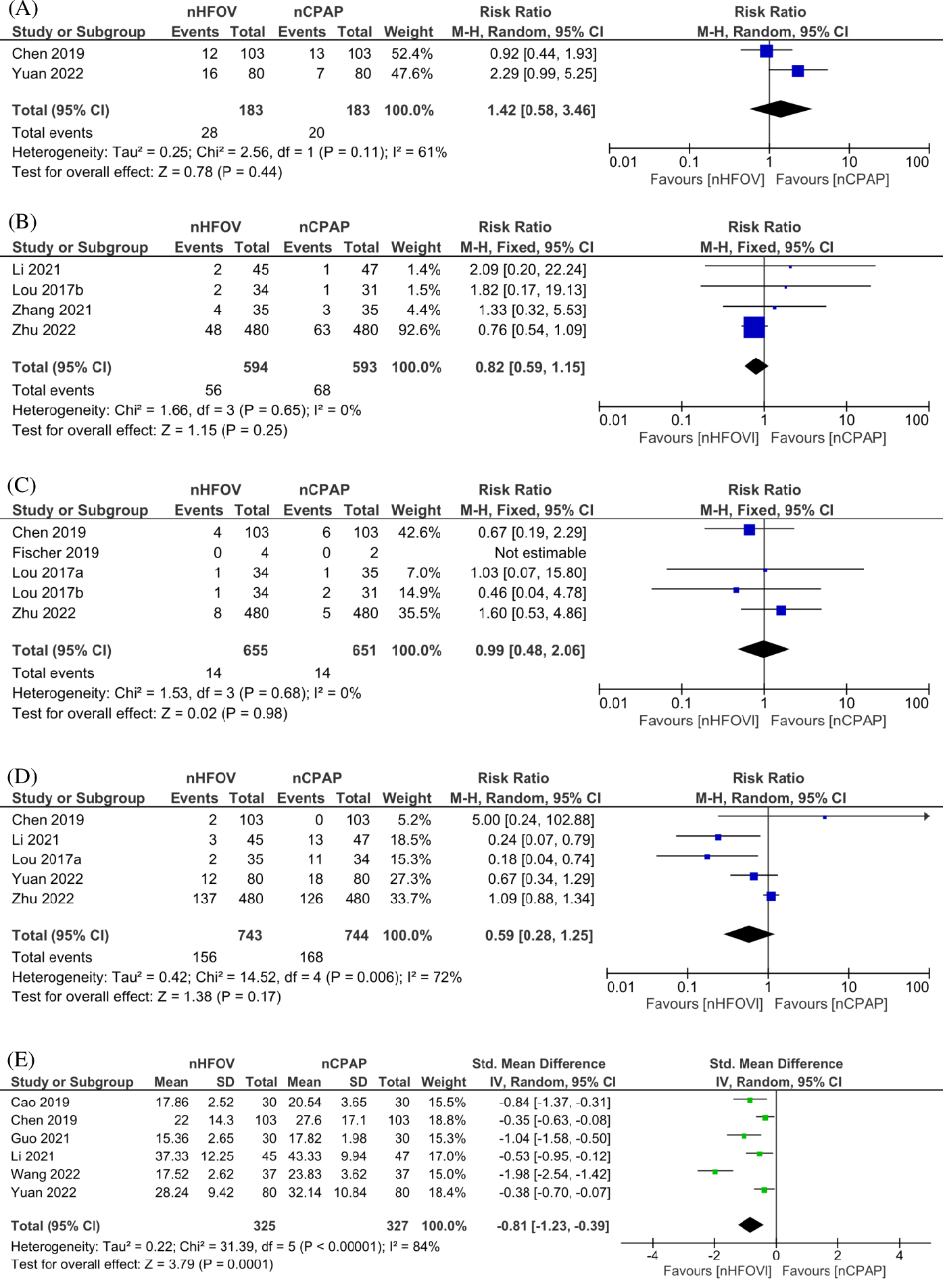

Supplement: S5 Fig — Forest plot of comparison: NHFOV vs NCPAP, outcome: (A) Intraventricular haemorrhage, any grade; (B) Intraventricular haemorrhage, grade ⪰3; (C) All-cause mortality (before hospital discharge); (D) Nasal injury; (E) Length of hospital stay, days. (TIF) [file pone.0307903.s007.tif]

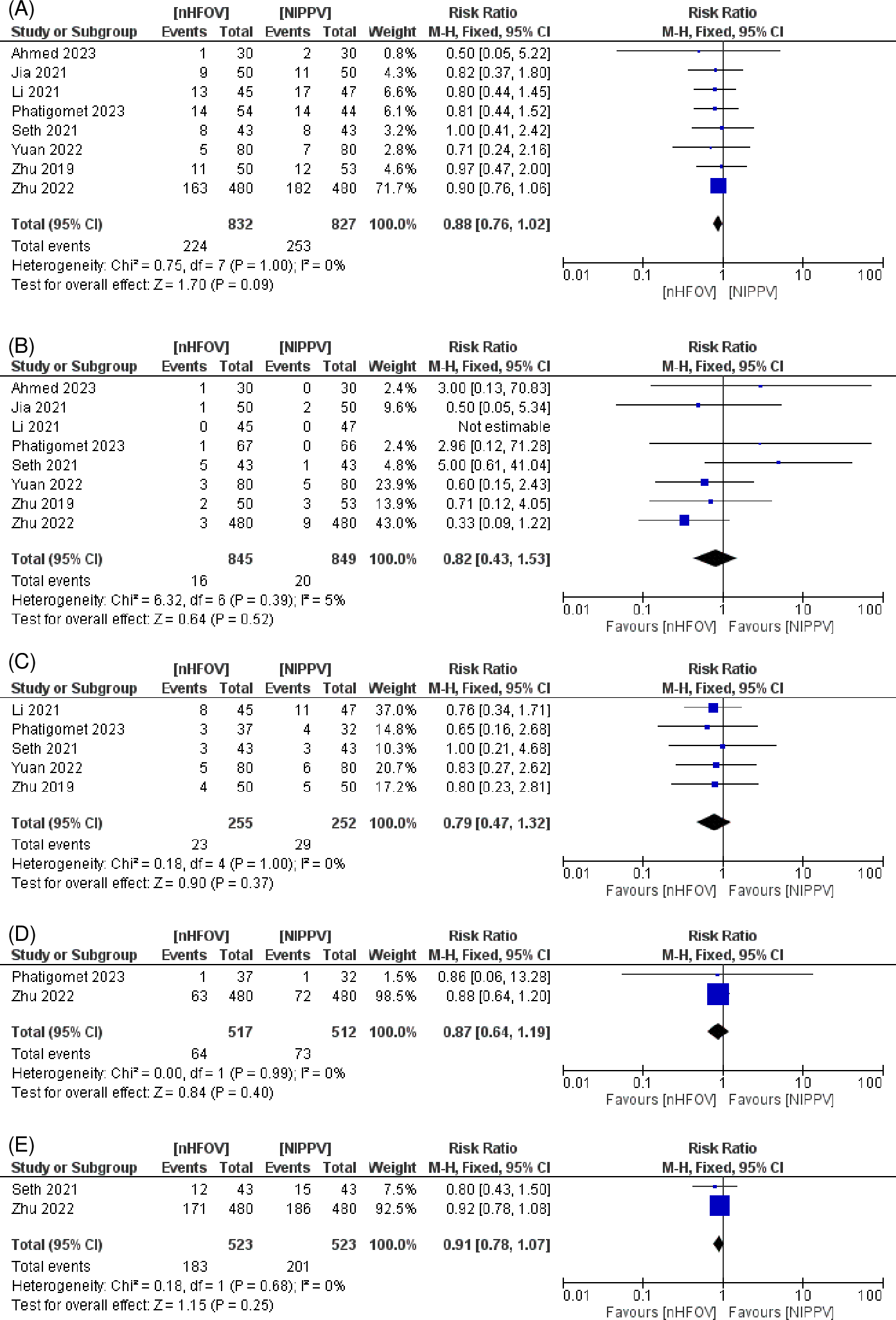

Supplement: S6 Fig — Forest plot of comparison: NHFOV vs NIPPV, outcome: (A) Bronchopulmonary dysplasia; (B) Pulmonary air leak; (C) Retinopathy of prematurity, any stage; (D) Retinopathy of prematurity, severe stage ≥3; (E) Composite outcome of death/BPD. (TIF) [file pone.0307903.s008.tif]

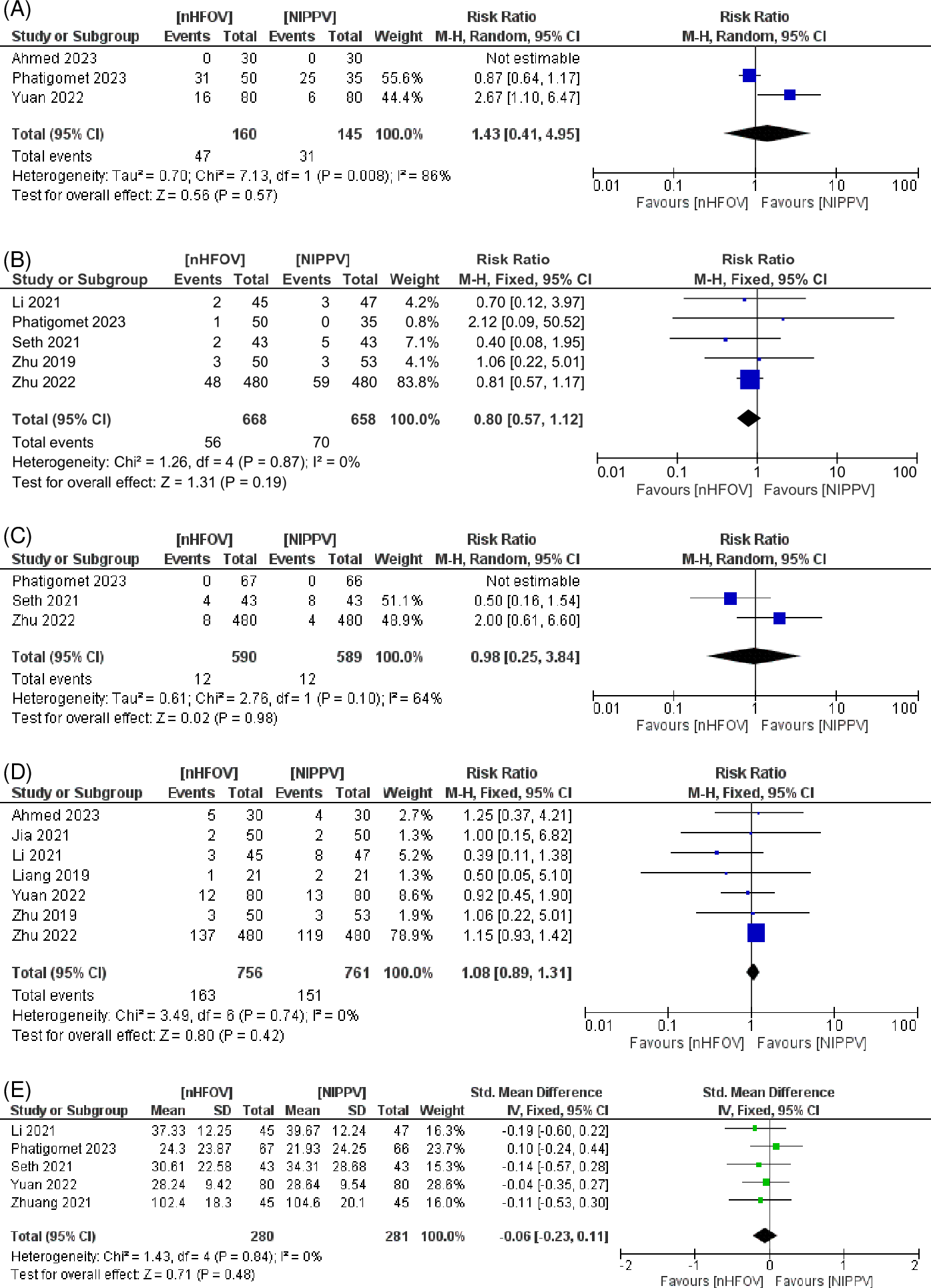

Supplement: S7 Fig — Forest plot of comparison: NHFOV vs NIPPV, outcome: (A) Intraventricular haemorrhage, any grade; (B) Intraventricular haemorrhage, grade ⪰3; (C) All-cause mortality (before hospital discharge); (D) Nasal injury; (E) Length of hospital stay, days. (TIF) [file pone.0307903.s009.tif]

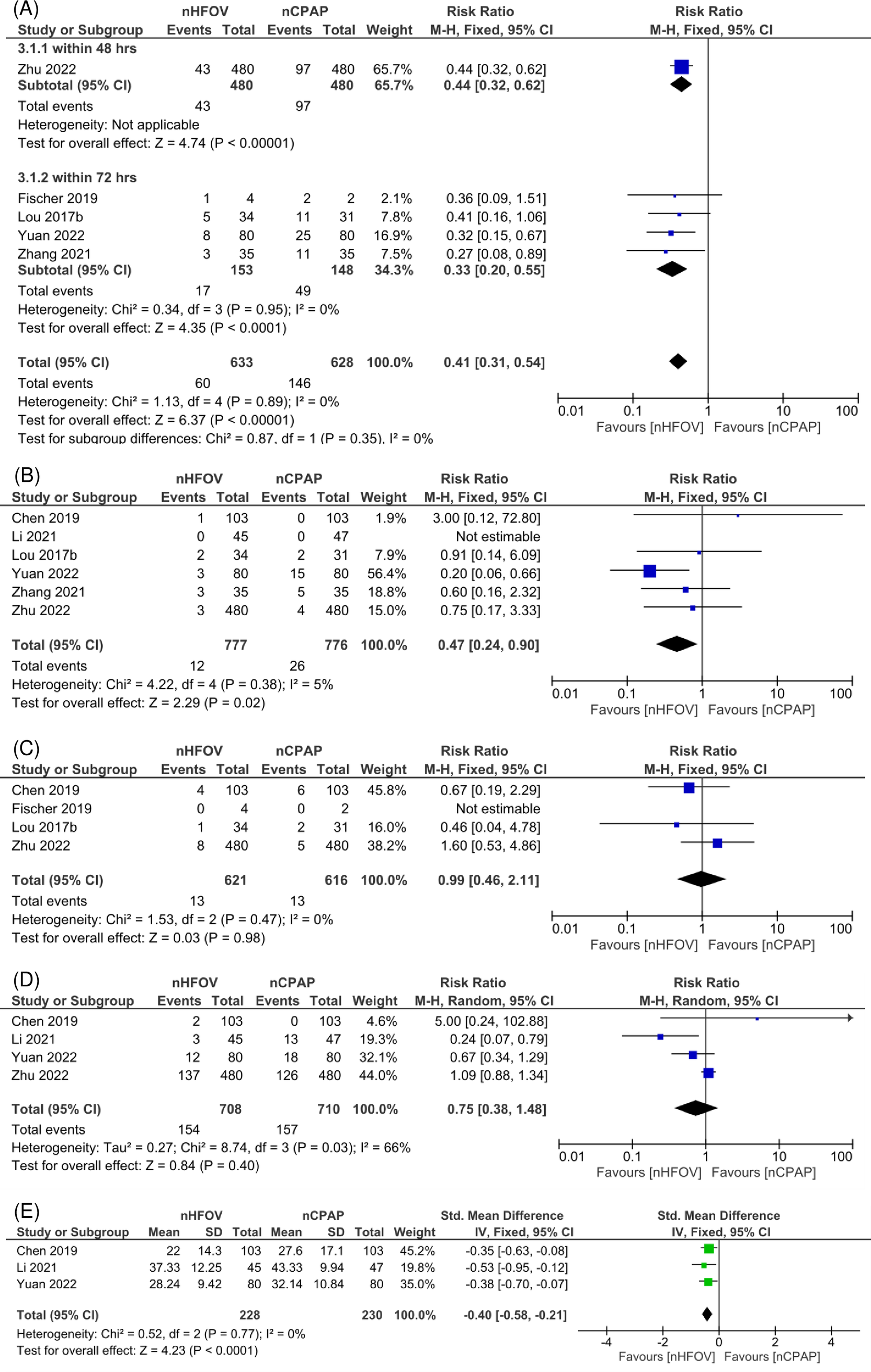

Supplement: S8 Fig — Forest plot of comparison: NHFOV vs NCPAP (preterm subgroup), outcome: (A) Extubation failure; (B) Pulmonary air leak; (C) All-cause mortality (before hospital discharge); (D) Nasal injury; (E) Length of hospital stay, days. (TIF) [file pone.0307903.s010.tif]

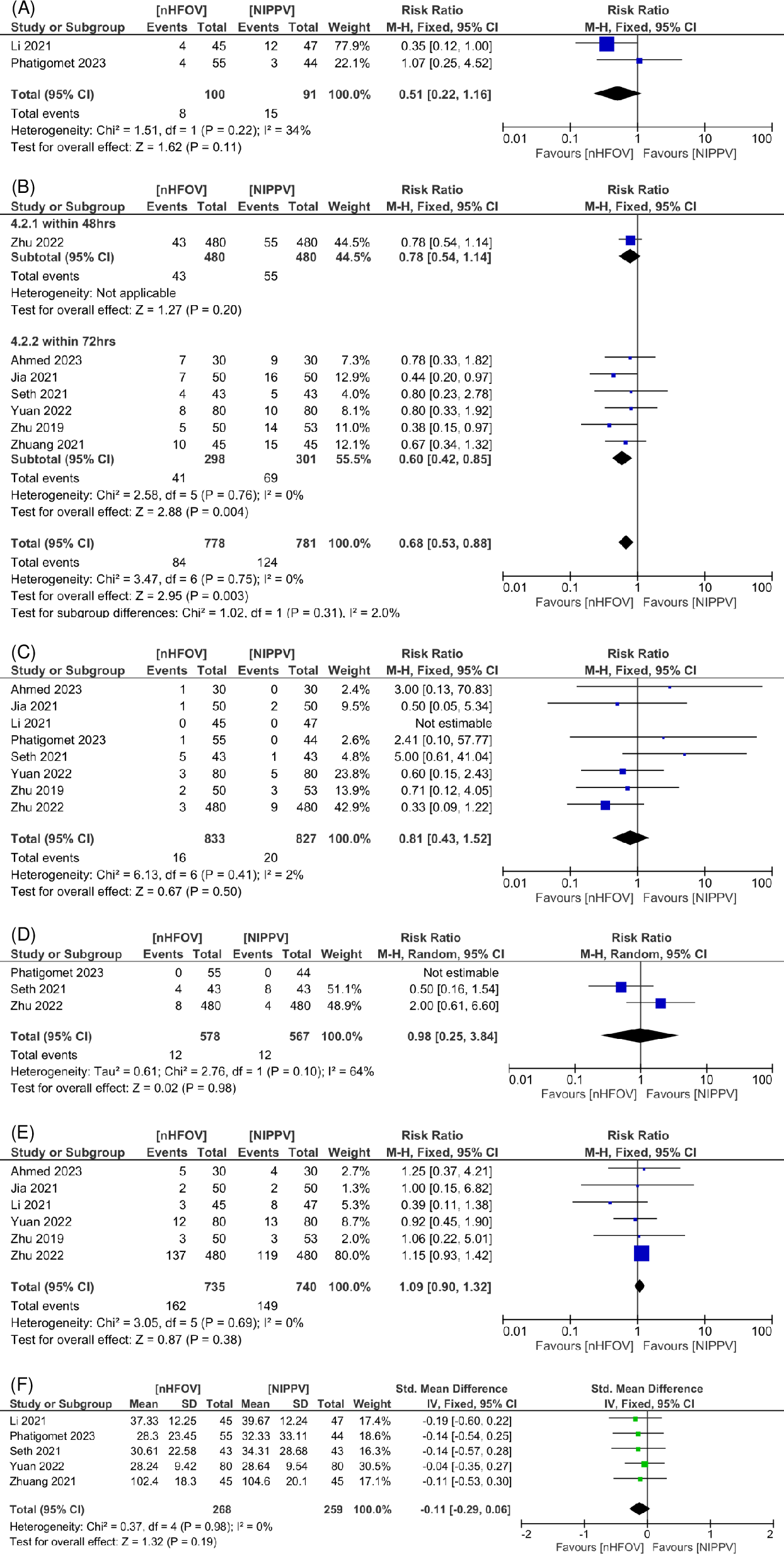

Supplement: S9 Fig — Forest plot of comparison: NHFOV vs NIPPV (preterm subgroup), outcome: (A) Reintubation (within 7 days of extubation); (B) Extubation failure; (C) Pulmonary air leak; (D) All-cause mortality (before hospital discharge); (E) Nasal injury; (F) Length of hospital stay, days. (TIF) [file pone.0307903.s011.tif]

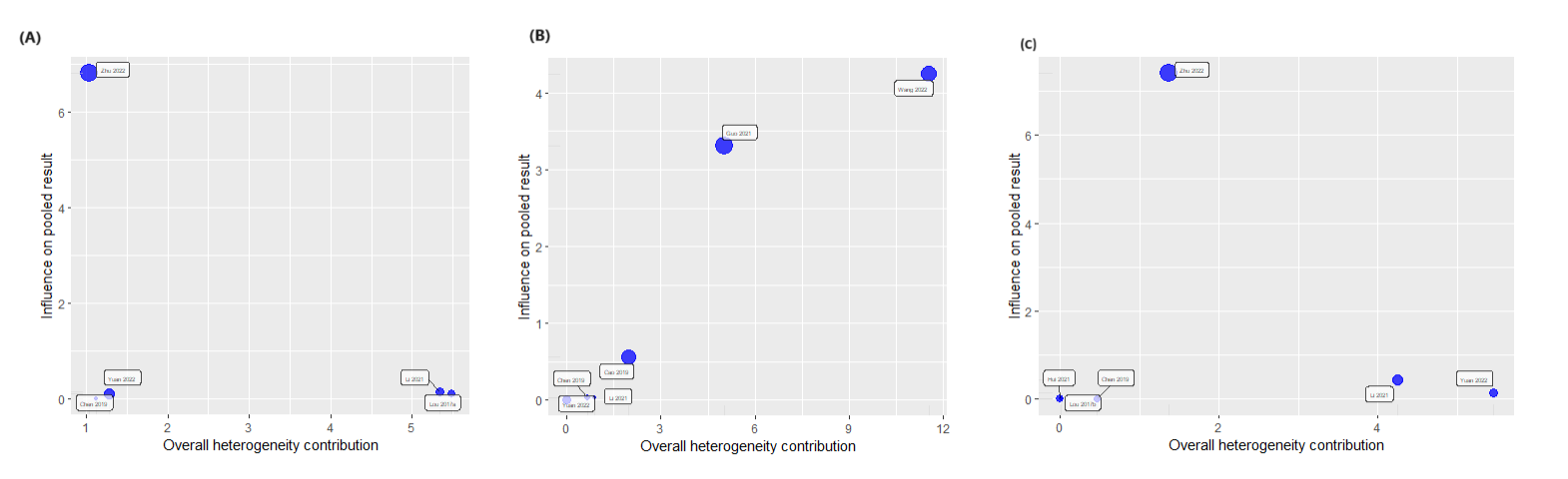

Supplement: S10 Fig — Baujat plot showing the studies that contributed to heterogeneity of comparison NHFOV vs NCPAP, outcome: (A) Bronchopulmonary dysplasia; (B) Nasal injury; (C) Length of hospital stay, days. (TIF) [file pone.0307903.s012.tif]

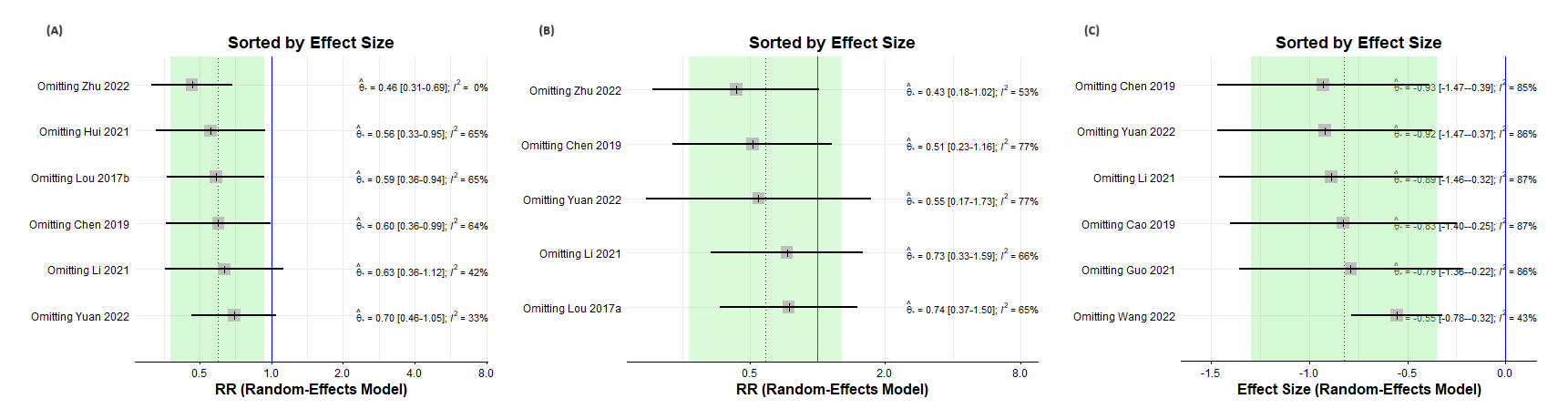

Supplement: S11 Fig — Leave-one-out meta-analysis: NHFOV vs NCPAP, outcome: (A) Bronchopulmonary dysplasia; (B) Nasal injury; (C) Length of hospital stay. (TIF) [file pone.0307903.s013.tif]

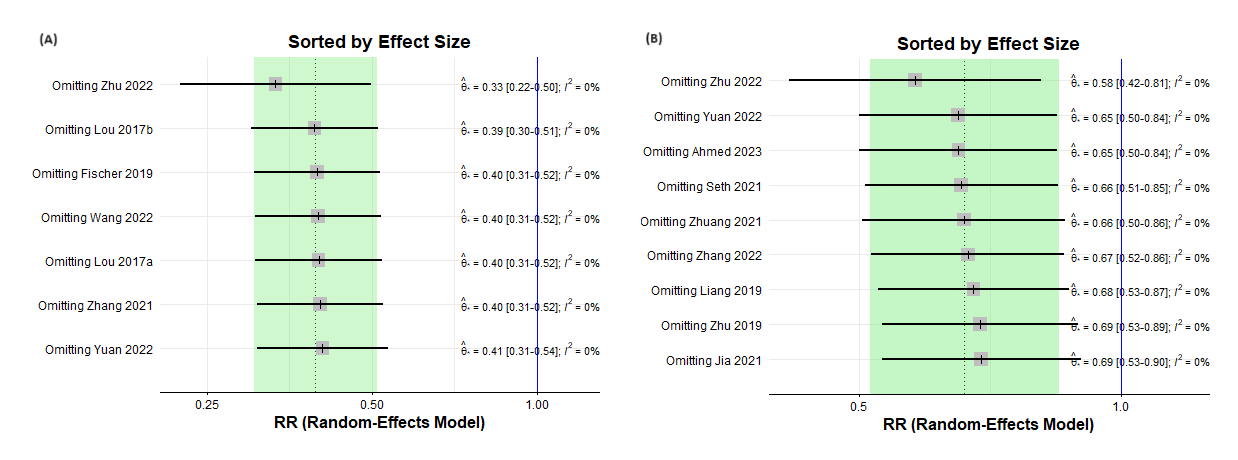

Supplement: S12 Fig — Leave-one-out meta-analysis of Extubation failure, comparison: (A) NHFOV vs NCPAP; (B) NHFOV vs NIPPV. (TIF) [file pone.0307903.s014.tif]

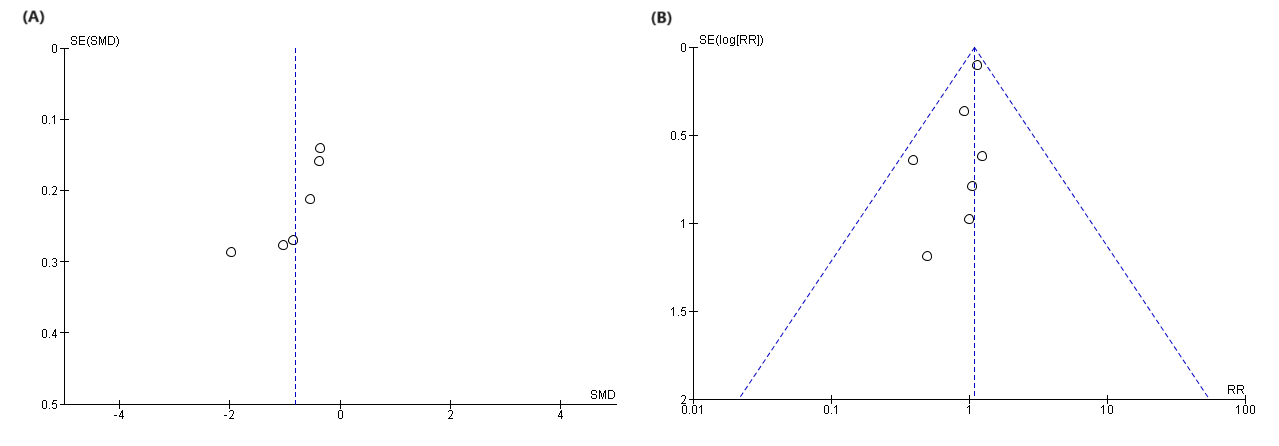

Supplement: S13 Fig — Funnel plot showing publication bias: (A) comparison NHFOV vs NCPAP, outcome: Length of hospital stay; (B) comparison NHFOV vs NIPPV, outcome: Nasal injury. (TIF) [file pone.0307903.s015.tif]
