# Supplementary material for: Noninvasive high-frequency oscillation ventilation as post- extubation respiratory support in neonates: Systematic review and meta-analysis
Source: PLoS One. 2024 Jul 30;19(7):e0307903. doi: 10.1371/journal.pone.0307903 (PMC11288463; doi:10.1371/journal.pone.0307903)
Supplement: S1 Table — (DOCX) [file pone.0307903.s016.docx]

**S1 Table. Search strategy**

1. **MEDLINE (PubMed)**

Date of Search: 30 June 2023

Number of results: 304

| **#** | **Search string** | **Results** |
| --- | --- | --- |
| 1 | ("infant, newborn"[Mesh] OR "Intensive Care, Neonatal"[Mesh] OR "Intensive Care Units, Neonatal"[Mesh] OR "Gestational Age"[Mesh]) OR (newborn* OR "new born" OR "new borns" OR "newly born" OR baby*[TIAB] OR babies[TIAB] OR premature OR prematures OR prematurity OR preterm OR preterms OR "pre term" OR preemie* OR premie* OR "low birth weight" OR "low birthweight" OR vlbw[TIAB] OR lbw[TIAB] OR neonat*[TIAB] OR infan*[TIAB]) | 14,42,040 |
| 2 | ("randomized controlled trial"[Publication Type] OR "controlled clinical trial"[Publication Type] OR "randomized"[Title/Abstract] OR "placebo"[Title/Abstract] OR "drug therapy"[MeSH Subheading] OR "randomly"[Title/Abstract] OR ("trial"[Title/Abstract] OR "groups"[Title/Abstract])) NOT ("animals"[MeSH Terms] NOT "humans"[MeSH Terms]) | 50,48,910 |
| 3 | "High-Frequency Ventilation"[Mesh] OR ((High frequency) AND (ventilat* OR oscillat*)) OR ((High-Frequency) AND (Ventilat* OR Oscillat*)) OR HFV OR HFO OR HFOV OR High frequency respirat* OR Oscillat* ventilation | 70,522 |
| 4 | non-invasive OR noninvasive OR non invasive OR nasal OR nasopharyngeal | 6,12,161 |
| 5 | #3 AND #4 | 5,457 |
| 6 | #1 AND #2 AND #5 | 304 |

(sensitivity-maximizing version of the Cochrane Highly Sensitive Search Strategy (verson 2008, was used for RCTs)

1. **Database name: CENTRAL**

Date of Search: 30 June 2023

Number of results: 178

| **#** | **Search string** | **# of results** |
| --- | --- | --- |
| 1 | infant or infants or infant’s or “infant s” or infantile or infancy or newborn* or "new born" or "new borns" or "newly born" or neonat* or baby* or babies or premature or prematures or prematurity or preterm or preterms or "pre term" or premies or "low birth weight" or "low birthweight" or VLBW or LBW or ELBW or NICU | 106960 |
| 2 | high frequency ventilat* | 2091 |
| 3 | high frequency oscillat* | 997 |
| 4 | Oscillatory ventilat* | 392 |
| 5 | HFV OR HFO OR HFOV | 388 |
| 6 | #2 OR #3 OR #4 OR #5 | 2735 |
| 7 | non-invasive OR noninvasive OR non invasive OR nasal OR nasopharyngeal | 55562 |
| 8 | #6 AND #7 | 815 |
| 9 | #1 AND #8 in Trials | 178 |

1. **Database name: Clinicaltrials.gov**

Date of Search: 30-June-2023

Number of results: 33

| **#** | **Search string** | **# of results** |
| --- | --- | --- |
| 1 | **Advanced Search**  **Intervention/Treatment**  (high frequency ventilation OR high-frequency ventilation OR ((high frequency OR high-frequency) AND (oscillatory OR oscillation OR oscillator)) OR HFOV OR HFO) AND (nasal OR noninvasive OR non-invasive OR non invasive)  Applied Filters : Child (birth–17) | 33 |

1. **Database name: WHO ICTRP**

Date of Search: 30 June 2023

Number of results: 51

| **#** | **Search string** | **# of results** |
| --- | --- | --- |
| 1 | **Advanced search**  **Look for trials with the exact phrase or contains**  high frequency ventilation OR high-frequency ventilation OR high frequency oscillat* OR high-frequency oscillat* OR HFOV OR HFO  **(in the intervention)**  **Applied Filters:** Search for clinical trials in children  Recruitment status is: All | 51 |

1. **Database name: Chinese National Knowledge Infrastructure (CNKI)**

Date of Search: 30 June 2023

Number of results: 92

| **#** | **Search string** | **# of results** |
| --- | --- | --- |
| 1 | **Professional search**  SU = ('high-frequency'+'high frequency') AND SU = ('noninvasive'+'non-invasive')  Applied filters: Cross-Language Search | 92 |
