# Supplementary material for: Noninvasive high-frequency oscillation ventilation as post- extubation respiratory support in neonates: Systematic review and meta-analysis
Source: PLoS One. 2024 Jul 30;19(7):e0307903. doi: 10.1371/journal.pone.0307903 (PMC11288463; doi:10.1371/journal.pone.0307903)
Supplement: S2 Table — (DOCX) [file pone.0307903.s017.docx]

**S2 table: List of excluded studies**

| **S no.** | **Reports** | **Reason for exclusion** |
| --- | --- | --- |
|  | NCT04905732 | Ongoing study (recruiting) |
|  | NCT02543125 | Status unknown, No response from author |
|  | NCT01852916 | Suspended (poor recruitment rate) |
|  | Mukherji 2016 [38] | Wrong indication |
|  | Li Huanhuan 2019[39] | Overlapping article, registration number: NCT03181958 |
|  | Lou 2018[40] | Wrong indication |
