# Supplementary material for: Noninvasive high-frequency oscillation ventilation as post- extubation respiratory support in neonates: Systematic review and meta-analysis
Source: PLoS One. 2024 Jul 30;19(7):e0307903. doi: 10.1371/journal.pone.0307903 (PMC11288463; doi:10.1371/journal.pone.0307903)
Supplement: S3 Table — (DOCX) [file pone.0307903.s018.docx]

| **S3 Table: NHFOV compared to NCPAP for respiratory support after extubation in neonates** | | | | | | | | | | | |
| --- | --- | --- | --- | --- | --- | --- | --- | --- | --- | --- | --- |
| **Certainty assessment** | | | | | | | **Summary of findings** | | | | |
| **Participants (studies) Follow-up** | **Risk of bias** | **Inconsistency** | **Indirectness** | **Imprecision** | **Publication bias** | **Overall certainty of evidence** | **Study event rates (%)** | | **Relative effect (95% CI)** | **Anticipated absolute effects** | |
|  |  |  |  |  |  |  | **With NCPAP** | **With NHFOV** |  | **Risk with NCPAP** | **Risk difference with NHFOV** |
| **Reintubation (within 7 days of extubation)** | | | | | | | | | | | |
| 304 (3 RCTs) | serious^a^ | not serious | not serious | serious^b^ | none | ⨁⨁◯◯ Low | 62/152 (40.8%) | 21/152 (13.8%) | **RR 0.34** (0.22 to 0.53) | 408 per 1,000 | **269 fewer per 1,000** (from 318 fewer to 192 fewer) |
| **Extubation failure within 72 hrs** | | | | | | | | | | | |
| 1404 (7 RCTs) | very serious^c^ | not serious | not serious | not serious | none | ⨁⨁◯◯ Low | 170/699 (24.3%) | 68/705 (9.6%) | **RR 0.39** (0.30 to 0.51) | 243 per 1,000 | **148 fewer per 1,000** (from 170 fewer to 119 fewer) |
| **Bronchopulmonary dysplasia** | | | | | | | | | | | |
| 1553 (6 RCTs) | serious^d^ | serious^e^ | not serious | not serious | none | ⨁⨁◯◯ Low | 245/776 (31.6%) | 190/777 (24.5%) | **RR 0.59** (0.37 to 0.94) | 316 per 1,000 | **129 fewer per 1,000** (from 199 fewer to 19 fewer) |
| **Pulmonary air leak** | | | | | | | | | | | |
| 1896 (11 RCTs) | serious^d^ | not serious | not serious | not serious | none | ⨁⨁⨁◯ Moderate | 38/947 (4.0%) | 17/949 (1.8%) | **RR 0.46** (0.27 to 0.79) | 40 per 1,000 | **22 fewer per 1,000** (from 29 fewer to 8 fewer) |
| **All-cause mortality (before hospital discharge)** | | | | | | | | | | | |
| 1306 (5 RCTs) | serious^d^ | not serious | not serious | very serious^f^ | none | ⨁◯◯◯ Very low | 14/651 (2.2%) | 14/655 (2.1%) | **RR 0.99** (0.48 to 2.06) | 22 per 1,000 | **0 fewer per 1,000** (from 11 fewer to 23 more) |
| **Retinopathy of prematurity, severe stage ≥3** | | | | | | | | | | | |
| 960 (1 RCT) | serious^a^ | not serious | not serious | serious^g^ | none | ⨁⨁◯◯ Low | 74/480 (15.4%) | 63/480 (13.1%) | **RR 0.85** (0.62 to 1.16) | 154 per 1,000 | **23 fewer per 1,000** (from 59 fewer to 25 more) |
| **Intraventricular haemorrhage, grade ⪰3** | | | | | | | | | | | |
| 1187 (4 RCTs) | serious^d^ | not serious | not serious | serious^g^ | none | ⨁⨁◯◯ Low | 68/593 (11.5%) | 56/594 (9.4%) | **RR 0.82** (0.59 to 1.15) | 115 per 1,000 | **21 fewer per 1,000** (from 47 fewer to 17 more) |

**CI:** confidence interval; **RR:** risk ratio

#### Explanations

a. Downgraded one level for serious limitations based on: risk of bias (lack of blinding).

b. Downgraded one level for serious limitation based on: small sample size.

c. Downgraded two level for very serious limitations based on: risk of bias (lack of blinding, unclear risk of selection and reporting bias).

d. Downgraded one level for serious limitations based on: risk of bias (lack of blinding of participants and personnel, unclear risk of selection and reporting bias).

e. Downgraded one level for serious limitation based on: unexplained heterogeneity (I2=57%)

f. Downgraded two level for serious limitation based on: Wide CI includes clinically important benefit and harm

g. Downgraded one level for serious limitation based on: Wide CI includes clinically important benefit or harm
