# Supplementary material for: Noninvasive high-frequency oscillation ventilation as post- extubation respiratory support in neonates: Systematic review and meta-analysis
Source: PLoS One. 2024 Jul 30;19(7):e0307903. doi: 10.1371/journal.pone.0307903 (PMC11288463; doi:10.1371/journal.pone.0307903)
Supplement: S4 Table — (DOCX) [file pone.0307903.s019.docx]

| **S4 Table: NHFOV compared to NIPPV for respiratory support after extubation in neonates** | | | | | | | | | | | |
| --- | --- | --- | --- | --- | --- | --- | --- | --- | --- | --- | --- |
| **Certainty assessment** | | | | | | | **Summary of findings** | | | | |
| **Participants (studies) Follow-up** | **Risk of bias** | **Inconsistency** | **Indirectness** | **Imprecision** | **Publication bias** | **Overall certainty of evidence** | **Study event rates (%)** | | **Relative effect (95% CI)** | **Anticipated absolute effects** | |
|  |  |  |  |  |  |  | **With NIPPV** | **With NHFOV** |  | **Risk with NIPPV** | **Risk difference with NHFOV** |
| **Reintubation (within 7 days of extubation)** | | | | | | | | | | | |
| 225 (2 RCTs) | serious^a^ | not serious^b^ | not serious | very serious^c^ | none | ⨁◯◯◯ Very low | 16/113 (14.2%) | 9/112 (8.0%) | **RR 0.62** (0.18 to 2.14) | 142 per 1,000 | **54 fewer per 1,000** (from 116 fewer to 161 more) |
| **Extubation failure within 72 hrs** | | | | | | | | | | | |
| 1642 (9 RCTs) | very serious^d^ | not serious | not serious | serious^e^ | none | ⨁◯◯◯ Very low | 136/821 (16.6%) | 88/821 (10.7%) | **RR 0.65** (0.50 to 0.83) | 166 per 1,000 | **58 fewer per 1,000** (from 83 fewer to 28 fewer) |
| **Bronchopulmonary dysplasia** | | | | | | | | | | | |
| 1659 (8 RCTs) | serious^f^ | not serious | not serious | serious^e^ | none | ⨁⨁◯◯ Low | 253/827 (30.6%) | 224/832 (26.9%) | **RR 0.88** (0.76 to 1.02) | 1,129 per 1,000 | **136 fewer per 1,000** (from 271 fewer to 23 more) |
| **Pulmonary air leak** | | | | | | | | | | | |
| 1694 (8 RCTs) | serious^f^ | not serious | not serious | very serious^c^ | none | ⨁◯◯◯ Very low | 20/849 (2.4%) | 16/845 (1.9%) | **RR 0.82** (0.43 to 1.53) | 24 per 1,000 | **4 fewer per 1,000** (from 13 fewer to 12 more) |
| **All-cause mortality (before hospital discharge)** | | | | | | | | | | | |
| 1179 (3 RCTs) | serious^a^ | not serious^b^ | not serious | very serious^c^ | none | ⨁◯◯◯ Very low | 12/589 (2.0%) | 12/590 (2.0%) | **RR 0.98** (0.25 to 3.84) | 20 per 1,000 | **0 fewer per 1,000** (from 15 fewer to 58 more) |
| **Retinopathy of prematurity, severe stage ≥3** | | | | | | | | | | | |
| 1029 (2 RCTs) | serious^a^ | not serious | not serious | serious^e^ | none | ⨁⨁◯◯ Low | 73/512 (14.3%) | 64/517 (12.4%) | **RR 0.87** (0.64 to 1.19) | 143 per 1,000 | **19 fewer per 1,000** (from 51 fewer to 27 more) |
| **Intraventricular haemorrhage, grade ⪰3** | | | | | | | | | | | |
| 1326 (5 RCTs) | serious^f^ | not serious | not serious | serious^e^ | none | ⨁⨁◯◯ Low | 70/658 (10.6%) | 56/668 (8.4%) | **RR 0.80** (0.57 to 1.12) | 106 per 1,000 | **21 fewer per 1,000** (from 46 fewer to 13 more) |

**CI:** confidence interval; **RR:** risk ratio

#### Explanations

a. Downgraded one level for serious limitations based on: risk of bias (lack of blinding)

b. Not downgraded for inconsistency: heterogeneity can be explained by different inclusion criteria in the two studies.

c. Downgraded two level for serious limitation based on: Wide CI includes clinically important benefit and harm

d. Downgraded two level for very serious limitations based on: risk of bias (lack of blinding, unclear selection bias and reporting bias).

e. Downgraded one level for serious limitation based on: Wide CI includes clinically important benefit or harm

f. Downgraded one level for serious limitations based on: risk of bias (lack of blinding of participants and personnel, unclear risk of selection and reporting bias
