# Supplementary material for: Noninvasive high-frequency oscillation ventilation as post- extubation respiratory support in neonates: Systematic review and meta-analysis
Source: PLoS One. 2024 Jul 30;19(7):e0307903. doi: 10.1371/journal.pone.0307903 (PMC11288463; doi:10.1371/journal.pone.0307903)
Supplement: S1 Appendix — (DOCX) [file pone.0307903.s024.docx]

**S1 Appendix**

**Data extraction**

Two authors independently extracted and recorded data from included studies on pre-designed form which was piloted initially on a few studies. Disagreements between the two reviewers were resolved by discussion or consensus with a third reviewer.

We extracted the characteristics of each study. Author, year of publication, study design, characteristics of the study population, sample size, noninvasive ventilation mode, the number of participants in intervention groups, noninvasive ventilator parameters, and outcomes of the included studies and details related to the methodological quality were recorded. We attempted to contact the author of the original report to obtain further details when any relevant information was missing.

**Assessment of heterogeneity**

The pooled forest plots were visually evaluated for assessing heterogeneity. Furthermore, we used the Chi‐ square test (χ^2^ test) and I^2^ statistics to assess heterogeneity. When the p > 0.1 or I^2^ was ≤50%, it was considered that there was no heterogeneity between trials.

**Investigation of heterogeneity**

If statistical heterogeneity was found then we planned to assess the source of the heterogeneity using subgroup analyses (gestational age, birth weight, RoB) if sufficient data was available.

**Publication Bias**

We examined publication bias for each meta-analysis, by visually examining asymmetry on funnel plots. Furthermore, asymmetry was examined using Egger's test at the 10% level if there were 10 or more studies.

**Results**

**RoB details**

**Selection bias**

Most Trials employed adequate methods to generate random sequences. Most studies used random number table [23, 28-32, 34-36, 41, 42] or computer-generated random sequence [17, 19, 20, 22, 24, 40]. Four trials did not describe the sequence generation process [25-27, 33]. Six trials employed adequate methods to ensure allocation concealment by sealed opaque envelopes or by using a dedicated and secured website and concealing the sequence from investigators [17, 19, 20, 22-24]. Other trials provided no or insufficient information on allocation concealment [25-36, 40-42].

**Performance bias**

All studies were deemed to have a high RoB due to the absence of blinding participants and personnel, and this bias was inevitable due to the nature of the intervention. Most of the studies did not provide information on blinding.

Due to the nature of the intervention, blinding to the caregivers was impossible and blinding of the neonates was not relevant. If blinding was not reported, we judged that blinding was not done due to the nature of the intervention. We assessed all trials at high risk of performance bias.

**Detection bias**

Only two studies blinded outcome assessors[17, 22]. Other studies were judged as at unclear RoB as no information was provided.

**Incomplete outcome data (Attrition bias)**

All studies reported complete or near-complete data for all randomized participants. We judged attrition bias to be low for all trials.

**Selective reporting**

We identified trial protocols in seven studies. We judged these studies as at low RoB. One study[33] was judged as at high RoB. Unclear extubation failure criteria prevented its inclusion in the meta-analysis. Reporting bias was unclear in other studies due to lack of availability of a protocol with pre‐specified outcomes.

**Other sources of bias**

One study was judged as at high RoB[33]. The participants were mostly term neonates and they received porcine surfactant before intubation. This intervention was likely inappropriate.

**Amendments in protocol:** We included CNKI database to broaden the search. We removed secondary outcome “need for additional ventilatory support within 7 days post extubation” as we found only few studies reported this and it was deemed clinically less important by our review team.
