# Supplementary material for: Noninvasive high-frequency oscillation ventilation as post- extubation respiratory support in neonates: Systematic review and meta-analysis
Source: PLoS One. 2024 Jul 30;19(7):e0307903. doi: 10.1371/journal.pone.0307903 (PMC11288463; doi:10.1371/journal.pone.0307903)
Supplement: Supplementary file 13 [file pone.0307903.s020.odt]

S5 Table: NHFOV versus NCPAP: subgroup analysis for preterm

| Outcome | Number of studies | Participants | Effect Estimate, RR [95% CI] | Population |
| --- | --- | --- | --- | --- |
| Reintubation (within 7 days of extubation) | 3 | 304 | 0.34 [0.22, 0.53] | preterm |
| Extubation failure | 7 | 1404 | 0.39 [0.30, 0.51] | all neonates |
| Extubation failure | 5 | 1261 | 0.41 [0.31, 0.54] | preterm |
| Bronchopulmonary dysplasia (BPD) | 6 | 1553 | 0.59 [0.37, 0.94] | preterm |
| Composite outcome of death/BPD | 2 | 966 | 0.90 [0.77, 1.06] | preterm |
| Pulmonary air leak | 11 | 1896 | 0.46 [0.27, 0.79] | all neonates |
| Pulmonary air leak | 6 | 1553 | 0.47 [0.24, 0.90] | preterm |
| All-cause mortality (before hospital discharge) | 5 | 1306 | 0.99 [0.48, 2.06] | all neonates |
| All-cause mortality (before hospital discharge) | 4 | 1237 | 0.99 [0.46, 2.11] | preterm |
| Retinopathy of prematurity, any stage | 3 | 458 | 0.55 [0.25, 1.20] | preterm |
| Retinopathy of prematurity, severe stage    ≥3 | 1 | 960 | 0.85 [0.62, 1.16] | preterm |
| Intraventricular haemorrhage, any grade | 2 | 366 | 1.42 [0.58, 3.46] | preterm |
| Intraventricular haemorrhage, grade ⪰3 | 4 | 1187 | 0.82 [0.59, 1.15] | preterm |
| Nasal injury | 5 | 1487 | 0.59 [0.28, 1.25] | all neonates |
| Nasal injury | 4 | 1418 | 0.75 [0.38, 1.48] | preterm |
| Length of hospital stay, days | 6 | 652 | -0.81 [-1.23, -0.39]* | all neonates |
| Length of hospital stay, days | 3 | 458 | -0.40 [-0.58, -0.21]* | preterm |

RR, risk ratio; CI, confidence interval.

*Standardized mean difference (95% Confidence Interval)
