# Supplementary material for: Noninvasive high-frequency oscillation ventilation as post- extubation respiratory support in neonates: Systematic review and meta-analysis
Source: PLoS One. 2024 Jul 30;19(7):e0307903. doi: 10.1371/journal.pone.0307903 (PMC11288463; doi:10.1371/journal.pone.0307903)
Supplement: Supplementary file 14 [file pone.0307903.s021.odt]

S6 Table: NHFOV versus NIPPV: subgroup analysis for preterm neonates 

| Outcome | Number of studies | Participants | Effect Estimate, RR [95% CI] | Subgroup |
| --- | --- | --- | --- | --- |
| Reintubation (within 7 days of extubation) | 2 | 225 | 0.62 [0.18, 2.14] | all neonates |
| Reintubation (within 7 days of extubation) | 2 | 191 | 0.51 [0.22, 1.16] | preterm |
| Extubation failure | 9 | 1642 | 0.65 [0.50, 0.83] | all neonates |
| Extubation failure | 7 | 1559 | 0.68 [0.53, 0.88] | preterm |
| Bronchopulmonary dysplasia (BPD) | 8 | 1659 | 0.88 [0.76, 1.02] | preterm |
| Composite outcome of death/BPD | 2 | 1046 | 0.91 [0.78, 1.07] | preterm |
| Pulmonary air leak | 8 | 1694 | 0.82 [0.43, 1.53] | all neonates |
| Pulmonary air leak | 8 | 1660 | 0.81 [0.43, 1.52] | preterm |
| All-cause mortality (before hospital discharge) | 3 | 1179 | 0.98 [0.25, 3.84] | all neonates |
| All-cause mortality (before hospital discharge) | 3 | 1145 | 0.98 [0.25, 3.84] | preterm |
| Retinopathy of prematurity, any stage | 5 | 507 | 0.79 [0.47, 1.32] | preterm |
| Retinopathy of prematurity, severe stage    ≥3 | 2 | 1029 | 0.87 [0.64, 1.19] | preterm |
| Intraventricular haemorrhage,any grade | 3 | 305 | 1.43 [0.41, 4.95] | preterm |
| Intraventricular haemorrhage, grade ⪰3 | 5 | 1326 | 0.80 [0.57, 1.12] | preterm |
| Nasal injury | 7 | 1517 | 1.08 [0.89, 1.31] | all neonates |
| Nasal injury | 6 | 1475 | 1.09 [0.90, 1.32] | preterm |
| Length of hospital stay, days | 5 | 561 | -0.06 [-0.23, 0.11]* | all neonates |
| Length of hospital stay, days | 5 | 527 | -0.11 [-0.29, 0.06]* | preterm |

RR, risk ratio; CI, confidence interval.

*Standardized mean difference (95% Confidence Interval)
