# Supplementary material for: Noninvasive high-frequency oscillation ventilation as post- extubation respiratory support in neonates: Systematic review and meta-analysis
Source: PLoS One. 2024 Jul 30;19(7):e0307903. doi: 10.1371/journal.pone.0307903 (PMC11288463; doi:10.1371/journal.pone.0307903)
Supplement: Supplementary file 15 [file pone.0307903.s022.odt]

S7 Table: NHFOV vs NCPAP, subgroup analysis for risk of bias

| Outcome or Subgroup | Number of studies | Participants | Effect Estimate, RR [95% CI] |
| --- | --- | --- | --- |
| Extubation failure | 7 | 1404 | 0.39 [0.30, 0.51] |
| Low risk of bias | 2 | 966 | 0.44 [0.32, 0.61] |
| High risk of bias | 5 | 438 | 0.33 [0.22, 0.51] |
| Bronchopulmonary dysplasia | 6 | 1553 | 0.59 [0.37, 0.94] |
| Low risk of bias | 3 | 1258 | 0.66 [0.39, 1.11] |
| High risk of bias | 3 | 295 | 0.48 [0.20, 1.14] |
| Pulmonary air leak | 11 | 1896 | 0.46 [0.27, 0.79] |
| Low risk of bias | 3 | 1258 | 1.00 [0.27, 3.68] |
| High risk of bias | 8 | 638 | 0.39 [0.21, 0.71] |
| All-cause mortality (before hospital discharge) | 5 | 1306 | 0.99 [0.48, 2.06] |
| Low risk of bias | 3 | 1172 | 1.09 [0.49, 2.44] |
| High risk of bias | 2 | 134 | 0.64 [0.11, 3.67] |
| Intraventricular haemorrhage, grade ⪰3 | 4 | 1187 | 0.82 [0.59, 1.15] |
| Low risk of bias | 2 | 1052 | 0.78 [0.55, 1.11] |
| High risk of bias | 2 | 135 | 1.46 [0.43, 4.92] |
| Nasal injury | 5 | 1487 | 0.59 [0.28, 1.25] |
| Low risk | 3 | 1258 | 0.78 [0.22, 2.81] |
| High risk | 2 | 229 | 0.40 [0.11, 1.45] |
| Length of hospital stay, days | 6 | 652 | -0.81 [-1.23, -0.39]* |
| Low risk of bias | 2 | 298 | -0.41 [-0.64, -0.18]* |
| High risk of bias | 4 | 354 | -1.04 [-1.71, -0.36]* |

RR, risk ratio; CI, confidence interval.

*Standardized mean difference (95% Confidence Interval)
