# Supplementary material for: Noninvasive high-frequency oscillation ventilation as post- extubation respiratory support in neonates: Systematic review and meta-analysis
Source: PLoS One. 2024 Jul 30;19(7):e0307903. doi: 10.1371/journal.pone.0307903 (PMC11288463; doi:10.1371/journal.pone.0307903)
Supplement: Supplementary file 16 [file pone.0307903.s023.odt]

S8 Table: NHFOV vs NIPPV, subgroup analysis for risk of bias

| Outcome | Number of studies | Participants | Effect Estimate, RR [95% CI] |
| --- | --- | --- | --- |
| Extubation failure | 9 | 1642 | 0.65 [0.50, 0.83] |
| Low risk of bias | 2 | 1046 | 0.78 [0.55, 1.13] |
| High risk of bias | 7 | 596 | 0.54 [0.38, 0.76] |
| Bronchopulmonary dysplasia | 8 | 1659 | 0.88 [0.76, 1.02] |
| Low risk of bias | 4 | 1236 | 0.89 [0.76, 1.04] |
| High riks of bias | 4 | 423 | 0.83 [0.52, 1.33] |
| Pulmonary air leak | 8 | 1694 | 0.82 [0.43, 1.53] |
| Low risk of bias | 4 | 1271 | 0.90 [0.38, 2.16] |
| High risk of bias | 4 | 423 | 0.73 [0.29, 1.82] |
| Retinopathy of prematurity, any stage | 5 | 507 | 0.79 [0.47, 1.32] |
| Low risk of bias | 3 | 247 | 0.77 [0.41, 1.47] |
| High riks of bias | 2 | 260 | 0.82 [0.35, 1.91] |
| Intraventricular haemorrhage, grade ⪰3 | 5 | 1326 | 0.80 [0.57, 1.12] |
| Low riks of bias | 4 | 1223 | 0.79 [0.56, 1.11] |
| High riks of bias | 1 | 103 | 1.06 [0.22, 5.01] |
| Nasal injury | 7 | 1517 | 1.08 [0.89, 1.31] |
| Low risk of bias | 2 | 1052 | 1.10 [0.90, 1.36] |
| High risk of bias | 5 | 465 | 0.97 [0.57, 1.65] |
| Length of hospital stay, days | 5 | 561 | -0.06 [-0.23, 0.11]* |
| Low risk of bias | 3 | 311 | -0.05 [-0.28, 0.17]* |
| High risk of bias | 2 | 250 | -0.07 [-0.32, 0.18]* |

RR, risk ratio; CI, confidence interval.

*Standardized mean difference (95% Confidence Interval)
